# Supplementary material for: Residential green space and child intelligence and behavior across urban, suburban, and rural areas in Belgium: A longitudinal birth cohort study of twins
Source: PLoS Med. 2020 Aug 18;17(8):e1003213. doi: 10.1371/journal.pmed.1003213 (PMC7446904; doi:10.1371/journal.pmed.1003213)
Supplement: S1 STROBE Checklist — STROBE, strengthening the reporting of observational studies in epidemiology. (DOCX) [file pmed.1003213.s001.docx]

STROBE Statement—Checklist of items that should be included in reports of ***cohort studies***

|  | | Item No | Recommendation | Page No |
| --- | --- | --- | --- | --- |
| **Title and abstract** | | 1 | (*a*) Indicate the study’s design with a commonly used term in the title or the abstract | (a) In Title: ‘a longitudinal birth cohort study of twins’. |
|  |  |  | (*b*) Provide in the abstract an informative and balanced summary of what was done and what was found | (b) In the Methods and Findings section of Abstract. |
| Introduction | | | | |
| Background/rationale | | 2 | Explain the scientific background and rationale for the investigation being reported | In paragraph 1-5, Introduction. |
| Objectives | | 3 | State specific objectives, including any prespecified hypotheses | In paragraph 5, Introduction. |
| Methods | | | | |
| Study design | | 4 | Present key elements of study design early in the paper | In paragraph 1-2, of section ‘Subject Recruitment’, Methods . |
| Setting | | 5 | Describe the setting, locations, and relevant dates, including periods of recruitment, exposure, follow-up, and data collection | In paragraph 2, of section ‘Subject Recruitment’, Methods . |
| Participants | | 6 | (*a*) Give the eligibility criteria, and the sources and methods of selection of participants. Describe methods of follow-up | (a) In paragraph 2, of section ‘Subject Recruitment’, Methods. An overview is presented in the flowchart as supplement (S2 Fig). |
|  |  |  | (*b*) For matched studies, give matching criteria and number of exposed and unexposed | (b) / |
| Variables | | 7 | Clearly define all outcomes, exposures, predictors, potential confounders, and effect modifiers. Give diagnostic criteria, if applicable | In paragraph 1-2, of section ‘Data collection’, Methods.  In paragraph 1-2, of section ‘Green space and traffic related exposure’, Methods.  In paragraph 1-2, of section ‘Assessment of intelligence and behaviour during childhood, Methods. |
| Data sources/ measurement | | 8* | For each variable of interest, give sources of data and details of methods of assessment (measurement). Describe comparability of assessment methods if there is more than one group | In paragraph 1-2, of section ‘Data collection’, Methods.  In paragraph 1-2, of section ‘Green space and traffic related exposure’, Methods.  In paragraph 1-2, of section ‘Assessment of intelligence and behaviour during childhood, Methods. |
| Bias | | 9 | Describe any efforts to address potential sources of bias | In paragraph 2, of section ‘Subject Recruitment’, Methods: “We compared the characteristics of those that … “. |
| Study size | | 10 | Explain how the study size was arrived at | In paragraph 2, of section ‘Subject Recruitment’, Methods. An overview is presented in the flowchart as supplement (S2 Fig). |
| Quantitative variables | | 11 | Explain how quantitative variables were handled in the analyses. If applicable, describe which groupings were chosen and why | In paragraph 1-2, of section ‘Statistical analysis’, Methods. |
| Statistical methods | | 12 | (*a*) Describe all statistical methods, including those used to control for confounding | (a) In paragraph 1, of section ‘Statistical analysis’, Methods |
|  |  |  | (*b*) Describe any methods used to examine subgroups and interactions | (b) In paragraph 1, of section ‘Statistical analysis’, Methods: “We tested the interaction …”. |
|  |  |  | (*c*) Explain how missing data were addressed | (c) In paragraph 2, of section ‘Subject Recruitment’, Methods: “We excluded 14 twin pairs from our analysis …”. |
|  |  |  | (*d*) If applicable, explain how loss to follow-up was addressed | (d) In paragraph 2, of section ‘Subject Recruitment’, Methods. An overview is presented in the flowchart as supplement (S2 Fig). |
|  |  |  | (*e*) Describe any sensitivity analyses | (e) In paragraph 2, of section ‘Statistical analysis’, Methods. |
| Results | | | |  |
| Participants | | 13* | (a) Report numbers of individuals at each stage of study—eg numbers potentially eligible, examined for eligibility, confirmed eligible, included in the study, completing follow-up, and analysed | (a) In paragraph 2, of section ‘Subject Recruitment’, Methods. An overview is presented in the flowchart as supplement (S2 Fig). |
|  |  |  | (b) Give reasons for non-participation at each stage | (b) In paragraph 2, of section ‘Subject Recruitment’, Methods: “In total 64 mother/twin pairs …”. |
|  |  |  | (c) Consider use of a flow diagram | (c) Supplement (S2 Fig). |
| Descriptive data | | 14* | (a) Give characteristics of study participants (eg demographic, clinical, social) and information on exposures and potential confounders | (a) In paragraph 1, of section ‘Characteristics of the study population’, Results. |
|  |  |  | (b) Indicate number of participants with missing data for each variable of interest | (b) In paragraph 2, of section ‘Subject Recruitment’, Methods: “We excluded 14 twin pairs from our analysis …”. |
|  |  |  | (c) Summarise follow-up time (eg, average and total amount) | (c) In paragraph 2, of section ‘Subject Recruitment’, Methods. |
| Outcome data | | 15* | Report numbers of outcome events or summary measures over time | (a) In paragraph 1, of section ‘Characteristics of the study population’, Results. |
| Main results | 16 | (*a*) Give unadjusted estimates and, if applicable, confounder-adjusted estimates and their precision (eg, 95% confidence interval). Make clear which confounders were adjusted for and why they were included | | (a) In paragraph 1, of section ‘Intelligence in association with green space in childhood’, Results. These results are shown in Figure 1 and Figure 2. |
|  |  | (*b*) Report category boundaries when continuous variables were categorized | | (b) / |
|  |  | (*c*) If relevant, consider translating estimates of relative risk into absolute risk for a meaningful time period | | (c) / |
| Other analyses | 17 | Report other analyses done—eg analyses of subgroups and interactions, and sensitivity analyses | | In paragraph 4, of section ‘Intelligence in association with green space in childhood’, Results. |
| Discussion | | | | |
| Key results | 18 | Summarise key results with reference to study objectives | | In paragraph 1, Discussion. |
| Limitations | 19 | Discuss limitations of the study, taking into account sources of potential bias or imprecision. Discuss both direction and magnitude of any potential bias | | In paragraph 9, Discussion. |
| Interpretation | 20 | Give a cautious overall interpretation of results considering objectives, limitations, multiplicity of analyses, results from similar studies, and other relevant evidence | | In paragraph 1-9, Discussion. |
| Generalisability | 21 | Discuss the generalisability (external validity) of the study results | | In paragraph 8, Discussion. |
| Other information | | | | |
| Funding | 22 | Give the source of funding and the role of the funders for the present study and, if applicable, for the original study on which the present article is based | | Financial Disclosure Statement |

*Give information separately for exposed and unexposed groups.

**Note:** An Explanation and Elaboration article discusses each checklist item and gives methodological background and published examples of transparent reporting. The STROBE checklist is best used in conjunction with this article (freely available on the Web sites of PLoS Medicine at http://www.plosmedicine.org/, Annals of Internal Medicine at http://www.annals.org/, and Epidemiology at http://www.epidem.com/). Information on the STROBE Initiative is available at http://www.strobe-statement.org.
