# Supplementary material for: Residential green space and child intelligence and behavior across urban, suburban, and rural areas in Belgium: A longitudinal birth cohort study of twins
Source: PLoS Med. 2020 Aug 18;17(8):e1003213. doi: 10.1371/journal.pmed.1003213 (PMC7446904; doi:10.1371/journal.pmed.1003213)
Supplement: S1 Text — (PDF) [file pmed.1003213.s002.pdf]

Online questionnaire completed by mother of the twin

Last name and first name mother:
……………………………………………………………………………………………….

Email address (optional): ……………………………………………………….

Birth data twins: ……………………………………………………………………

First name child 1 (=first born twin):
………………………………………………………………………………………………

First name child 2 (=second born twin):
………………………………………………………………………………………………

If you have further comments concerning your personal information, you can write them down here. For example, an address change.
……………………………………………………………………………………….
……………………………………………………………………………………….
……………………………………………………………………………………….

What were the previous addresses of the children where they lived together? When did they live there? *We want to emphasize that the Data will be stored in a confidential way, in which your name will be replaced by a code.*

	Steet	Number	City	From (year)	Till (year)
Last address before pregnancy					
Address during pregnancy					
Birth address twin					
Address 2					
Address 3					
Address 4					
Address 5					
Address 6					

If you have further comments, you can write them down here.
……………………………………………………………………………………….
……………………………………………………………………………………….
……………………………………………………………………………………….

What is the highest diploma of your children? If your children following education at the moment and are in the process of obtaining a degree, you can indicate the future highest degree.

	Child 1	Child 2
**No Diploma**	❑	❑
**Primary education**	❑	❑
**Low secondary education (till 3th year)**	❑	❑
**Higher secondary education (till 6th year)**		
• BSO	❑	❑
• TSO or KSO	❑	❑
• ASO	❑	❑
**Higher education**		
• Higher education short type (max. 3 years)	❑	❑
• Higher education long type (4 years or more)	❑	❑
• University: Bachelor	❑	❑
• University: Master	❑	❑
**Other:** ……………………….	❑	❑

Which of the following situations are your children in at the moment?

	Child 1	Child 2
Full-time job	❑	❑
Part-time job	❑	❑
Student	❑	❑
Unemployed	❑	❑
Other: …………….	❑	❑
