# Supplementary material for: Residential green space and child intelligence and behavior across urban, suburban, and rural areas in Belgium: A longitudinal birth cohort study of twins
Source: PLoS Med. 2020 Aug 18;17(8):e1003213. doi: 10.1371/journal.pmed.1003213 (PMC7446904; doi:10.1371/journal.pmed.1003213)
Supplement: S2 Text — (PDF) [file pmed.1003213.s003.pdf]

Fonds de Soutien/steunfonds
Marguerite-Marie Delacroix

Residential surrounding greenness, traffic exposure and maternal stress as determinants of neurocognitive and neurobehavioural development during the first decade of life in twins and triplets

Environmental stressors and neurodevelopment of multiple births

Dr Esmée Bijnens

Promotors:

Prof. Dr Tim Nawrot

Prof. Dr Catherine Derom

Prof. Dr Evert Thiery

SUMMARY

Surrounding greenness at home, air pollution and maternal stress have been related to reduction of behavioural problems and improvement of cognitive development, but little is known about mechanism(s) underlying such associations. We hypothesize that exposure to residential greenness might affect *in utero* stress conditions, both social, as maternal perceived stress, as well as biological stress (inflammation) which both might play a role in behavioural and cognitive development in children. Our hypothesis is supported by available animal experiments showing cognitive effects of particulate air pollution exposure due to a pro-inflammatory state. There are very few human studies on cognitive development and residential traffic and greenness surrounding and non in a susceptible population segment such as twins and triplets.

In the first part of this project, neurocognition was assed among 663 twins pairs with a mean age of 10.4 years old by the Wechsler Intelligence Scale for Children-Revised (WISC-R) and among 752 twins pairs (mean age of 8.2 years), the neurobehavioural outcome was examined with the Child Behaviour Checklist (CBCL). Both test were performed by 52 triplet sets (140 children). In addition, placentation and perinatal data were recorded at birth.

In a second prospective part, momentary maternal stress during pregnancy will be assessed using The Experience Sampling Method (ESM), the Perceived Stress Scale and cortisol concentrations measured in hair. The association between maternal stress and residential greenness will be assessed. In addition, the intermediate role of maternal stress will be evaluated in the relationship between residential air pollution and biological marker of oxidative stress/inflammation. Greenness of the immediate home environment will be evaluated using detailed satellite-derived data, exposure to pre- and postnatal exposure will be assessed by use of land use and dispersion models.

The originality of our project include to study in a retrospective fashion, the association between exposure to greenness, traffic, and stress on the one hand and neurodevelopment on the other hand. In addition, in a prospective fashion, we will study perceived stress as a possible underlying mechanism. The project is relevant for society, indeed, the most recent EU key directive on ambient air quality is Directive 2008/50/EC. It recognizes the need to reduce pollution to levels, which minimize the harmful effects on human health. The limit values adopted for particulate matter are still higher than WHO recommends. For example, the annual average limit value for $PM_{2.5}$ was set at 25 μg/m^3, a level 2.5 times higher than recommended by WHO. The current project allows for detailed risk assessments considering interactions with a broad range of environmental exposures. The results of our study may lead to a better protection of the most vulnerable individuals in society, i.e. newborns, more specifically those with a complex pregnancy such as twins and triplets. We are seeing more twins now than ever before: twinning rates are at a peak, occurring in 1/30 births in western nations, double the 1980 rate of 1/60. The twins further allow to distinguish the impact of the genes and the environment on the neuropsychological tests and IQ. Knowing that genes, as well as environments, affect our abilities, talents and skills is crucial information for everyone to know. Further, the project might prioritize health related aspects more directly in the future urban planning.

SAMENVATTING

Een groen leefmilieu, blootstelling aan het verkeer en stress bij de moeder als determinanten van de neurocognitieve en de gedragsontwikkeling tijdens de eerste levensdecade van tweelingen en drielingen.

Recente studies hebben een groen leefmilieu en de afwezigheid van luchtvervuiling en van stress van de moeder tijdens de zwangerschap in verband gebracht met een reductie van gedragsproblemen en een verbetering van de cognitieve ontwikkeling. Over de mechanismen onderliggend aan deze associaties is weinig bekend.

We werken de hypothese uit dat veel groen in de leefomgeving en een reductie van de luchtvervuiling een duidelijk gunstig effect kunnen sorteren op stress tijdens de zwangerschap. Het betreft dan zowel sociale stress (met name die welke door de moeder wordt beleefd) als biologische stress (een ontstekingsproces), waarbij beide een rol lijken te spelen in de cognitieve en gedragsontwikkeling van het kind.

Deze hypothese wordt kracht bijgezet door dierexperimenten waarbij de cognitieve repercussie van luchtvervuiling via ontstekingsmechanismen werd aangetoond. Er zijn tot op heden nog maar weinig studies bij de mens en vooralsnog geen bij tweelingen en drielingen waarin de cognitieve ontwikkeling duidelijk medebepaald blijkt te zijn door de verkeerssituatie en de groene omgeving nabij de woonst in relatie tot stressmechanismen die reeds van voor de geboorte beeldbepalend lijken te zijn.

In een eerste studie wordt deze vraagstelling retrospectief bekeken bij meer dan 650 tweelingparen met een gemiddelde leeftijd van 10 jaar waarbij we beschikken over IQ gegevens en bij meer dan 750 tweelingparen met een gemiddelde leeftijd van 8 jaar waarbij we beschikken over gegevens betreffende de gedragsontwikkeling. Bovendien zijn zowel de gegevens over IQ als gedrag beschikbaar van 52 sets van drielingen (140 kinderen). Daarnaast, zijn van al deze kinderen placentatie- en perinatale gegevens voorhanden. Zodat markers van oxidatieve stress kunnen gemeten worden.

In een tweede prospectieve studie, wordt de tijdens de zwangerschap door de moeder beleefde stress nagegaan met de Perceived Stress Scale, de Experience Sampling Method en aan de hand van een meting van cortisol in het haar van de moeder. Naast het verband tussen groene ruimte en stress gaan we ook de intermediare rol van maternale stress bestuderen in de associatie tussen groen in de leefomgeving en moleculaire merkers van oxidatieve stress en ontsteking.

Groen in de nabije woonomgeving zal bepaald worden aan de hand van gedetailleerde data afkomstig van satellietbeelden. Blootstelling aan luchtvervuiling zowel tijdens de zwangerschap als na de geboorte zal bepaald op basis van landgebruik en dispersie modellen.

De originaliteit van het project is dat het op een retrospectieve manier, de associatie bestudeerd tussen enerzijds blootstelling aan groen, verkeer en stress en anderzijds neurobiologische

ontwikkeling. Bovendien zal op een prospectieve manier stress verder bestudeerd worden als mogelijk onderliggend mechanisme.

Dit project is relevant op maatschappelijk vlak want luchtvervuiling bedreigt onze gezondheid en de normen van de wereldgezondheidsorganisatie worden thans duidelijk overschreden. Ook laat dit project risicoanalyses toe op tal van toxische blootstellingen. Dit zal aanleiding geven tot een door allen ervaren nood aan betere protectie van de foetus, het pasgeboren en het opgroeiend kind, dankzij een studie van risicokinderen als tweelingen en drielingen die een complexe zwangerschapssituatie kennen. We weten bovendien dat het aantal tweelingen toeneemt, wat meteen het project nog relevanter maakt. Ook laten tweelingen toe het onderscheid te maken tussen de effecten van genen en omgeving op neuropsychologische testen en het IQ. Het is voor iedereen van belang om te weten dat zowel omgeving en genen van invloed zijn op onze talenten en vaardigheden. Tenslotte zal ons project ook implicaties hebben op de gezondheidszorg en de stedenbouwkundige planning van de toekomst.

RÉSUMÉ

Espaces verts, exposition à la circulation et stress maternel en tant que déterminants du développement neurocognitif et comportemental de jumeaux et triplés.

La proximité d'espaces verts, l'absence de pollution et de stress maternel pendant la grossesse ont été associés à la réduction de problèmes de comportement et à l'amélioration du développement cognitif de l'enfant. Par contre peu est connu sur les mécanismes sous-jacents de telles associations. Nous émettons l'hypothèse que la pollution et l'absence d'espaces verts peuvent influencer les conditions de stress « in utero » ; stress maternel et stress biologique (inflammation), qui à leur tour peuvent influencer le développement comportemental et cognitif de l'enfant. Notre hypothèse est étayée par des données animales disponibles démontrant des effets «cognitifs » dus à l'exposition de la pollution atmosphérique qui engendre un état pré-inflammatoire. Il n'existe aujourd'hui aucune étude de ce genre dans une population à risque telle que les jumeaux et triplés.

L'étude comprend 2 volets : un volet rétrospectif et un volet prospectif. L'étude rétrospective est composée de 2 études antérieures : 1) dans la première étude le développement neurocognitif a été évalué par un test de QI validé (le WISC-R) chez plus de 600 paires de jumeaux, de 10 ans d'âge en moyenne et 140 triplés (provenant de 52 sets de triplés) ; 2) dans la seconde étude le développement comportemental a été évalué par le « Child Behaviour Checklist » (CBCL) chez 752 paires de jumeaux, de 8 ans d'âge en moyenne et 140 triplés. La placentation ainsi que des données périnatales médicalement importantes ont été enregistrées à la naissance. Pour toutes ces naissances multiples, espaces verts et degré de pollution seront évalués à partir de données détaillées provenant de satellites et des modèles de dispersion et marqueurs biologiques du stress par la longueur des télomères et le contenu de l'ADN mitochondrial dans le tissu placentaire. Le stress maternel pendant la grossesse sera mesuré par le « Perceived Stress Scale » (PSS).

Dans le second volet (l'étude prospective) le stress maternel pendant la grossesse sera mesuré par le « Perceived Stress Scale » (PSS), la méthode ESM (Experience Sampling Method) et la concentration de cortisol, mesurée dans les cheveux. L'association entre le stress maternel, le degré de pollution et la proximité d'espaces verts sera évalué. En plus nous étudierons le rôle intermédiaire du stress maternel dans l'association entre le degré de pollution et les marqueurs biologiques du stress et de l'inflammation.

L'originalité de ce projet réside dans l'opportunité d'étudier d'une façon rétrospective (à l'aide de données récoltées dans des études antérieures) l'association entre le degré de pollution et le développement neurocognitif et comportemental de l'enfant et d'une façon prospective le rôle intermédiaire du stress maternel dans cette association. Ce projet est relevant pour la société et la santé publique. En effet, une des plus récentes directives clés de l'UE sur la qualité de l'air ambiant reconnaît la nécessité de réduire la pollution à des niveaux qui minimisent les effets nocifs sur la santé humaine. Nos résultats peuvent ainsi conduire à une meilleure protection des personnes les plus vulnérables de la société, les nouveau-nés, et plus particulièrement ceux qui ont une grossesse à risque telle que les jumeaux et triplés. Nous voyons plus de grossesses gémellaires que jamais: une naissance sur 30 dans les pays occidentaux, le double du taux de 1980 (1/60).

Finalement, l'étude par le biais des jumeaux permet en outre de distinguer l'impact des gènes et de l'environnement sur les interactions stress, pollution, espaces verts et le développement neurocognitif de l'enfant. Sachant comment l'environnement, en interaction avec nos gènes, affecte nos capacités, talents et compétences reste une information cruciale pour tout le monde. En outre, le projet pourrait donner des priorités dans la planification urbaine future.

INTRODUCTION

Greenness

Recently home surrounding greenness has been associated with a protective impact on emotional and behavioural problems in schoolchildren in Barcelona[1] which was also observed in a German birth cohort.[2] Both studies reported a larger decrease on hyperactivity and in attention symptoms. These findings were consistent with the effects of 'nature restoration' observed in a small sample of 17 children who improved their cognitive function after relocation to residences with higher 'naturalness'.[3] Accordingly, four trials have shown an improvement of attention deficit and hyperactivity disorder (ADHD) symptomatology after exposures to the natural environment.[4-6] Consistently, a recent US cross-sectional study has reported an improved school performance associated with school greenness based on data from all the schools in Massachusetts.[7] The mechanisms underlying observed associations between exposure to greenness and mental and psychological status are not well-understood.

Air pollution

Solid and liquid phase material suspended in the atmosphere is referred to as 'fine particulate matter' (PM). Cardiorespiratory effects and mechanism of particulate air pollution have been largely investigated.[8, 9] In contrast, little is known about neurological effects of fine particulate air pollution. There exist several pathways by which air pollution may exert neurotoxic effects. The direct ones results in an accumulation of ultrafine particles in het human brain.[10, 11] In rats, inhaled ultrafine particles translocate from the nose along the olfactory nerve to the olfactory bulb and other regions of the brain.[12-15] Additionally, studies in rodents have demonstrated that ultrafine particles translocate from the lungs into the blood, from which they can reach extrapulmonary organs including the brain.[16, 17] The indirect pathways involve a pro-inflammatory response in the lung when exposed to air pollution leading to increased systemic inflammation, which in turn, may adversely affect the brain.[18-22] Inflammatory mediators produced in the lung are able to translocate into the circulation[23] and trigger a sequel of pro-inflammatory events.[19, 24-26] Exposure to airborne polycyclic aromatic hydrocarbons (PAH) during the third trimester of pregnancy in women residing in New York.[27] Children with a prenatal PAHs exposure above the median had a 4.7 points (95% CI: -7.7 to -1.6) lower verbal IQ and 4.3 points (95% CI: -7.4 to -1.2) lower full scale IQ than those with exposure below the median.[27] The impact of PAHs on mental development at 3 years of age does not appear to be mediated by birth weight or head circumference, fetal growth parameters previously shown to be associated with prenatal PAH exposure in this cohort.[27, 28] These results are supported by a Polish study.[29] A PAH's exposure above the median assessed by personal air monitoring of the mother during the pregnancy, was associated with a 1.4 points decrease (95% CI: -2.5 to -0.2) in the Raven Progressive Matrices Test in a group of 214 five-year-old children.[29] A few studies investigated the association between long-term air pollution exposure and neurobehavioural performance in children. A study in Boston in 202 children with a mean age of 9.7 years showed that an interquartile-range increase in the lifetime residential black carbon concentrations was associated with a 3.4 points decrease in IQ (95% CI: -6.6 to -0.3) measured by the Kaufman Brief Intelligence Test and 3.9 points decrease (95% CI: -7.5 to -0.3) in a general index of the Wide Range Assessment

of Memory and Learning.[30] In addition, a study comparing cognitive performance of children living in clean areas and areas with much air pollution noted that children residing Mexico City performed more poorly on several subtests of the Wechsler Intelligence Scale for Children-Revised than controls matched for socioeconomic status.[31, 32]

The intermediate role of stress

We hypothesize that *in utero* stress conditions, both maternal perceived stress, as well as biological stress (inflammation and oxidative) might play an intermediate role between environmental exposure and cognitive development in children.

Studies observed a significant association between neighborhood green space and lower levels of symptomology for depression, anxiety and stress.[33, 34] In addition maternal stress during pregnancy is associated with adverse neurodevelopmental outcomes in the child. These include emotional or cognitive problems, such as increased risk of attentional deficit/hyperactivity, anxiety, and language delay.[35-37] However many questions remain about the underlying mechanism. A proposed mechanism by which prenatal stress may affect the fetus is cortisol.[38] During pregnancy, women have naturally elevated levels however under stressful conditions maternal cortisol concentrations can reach high levels. Consequently high concentrations of cortisol may affect fetal development and growth [39] and according to animal studies this may even alter the development of neurons in the brain.[40-42] The emotional state of the mother may also alter the function of the placenta in other ways, independent of cortisol. There is some evidence for effects on uterine blood supply, which could affect the neurodevelopment of the fetus.[43]

Besides maternal stress also biological stress, such as inflammation and oxidative stress, may serve as a mediator between environmental exposure and neurocognitive development. Exposure to particulate air pollution can result in oxidative stress and inflammation.[44, 45] This is supported by studies showing an association between *in utero* traffic exposure and shorter telomere length and decreased mitochondrial DNA content in placental tissue, both sensitive to the effects of oxidative stress.[46, 47] However the effects of biological stress during pregnancy on the development of the fetal brain and neurocognition in childhood are currently unknown.

Epigenetics to link the role of early life exposure with brain development

The placenta, aside from its role as exchange barrier, also contributes to neurodevelopmental processes through adaptive responses to the maternal environment. These critical developmental processes in the placenta and fetal brain are shaped by the same molecular signals.[48] Transcriptional changes during prenatal development are associated with morphological and functional development of the fetal brain.[49]

In mice, a strong correlation was observed between a set of genes, co-expressed in the hypothalamus and placenta at mid-gestation (embryonic day 11-13), an important period of neuronal proliferation and differentiation.[50] Bonnin *et al*. [51] showed in an ex vivo model that the placenta can convert

maternal tryptophan into the neurotransmitter serotonin (5-hydroxytryptamine; 5-HT) and functions as primary source of 5-HT for the developing mouse forebrain at mid-gestation.

Besides the serotenergic pathway, other pathways in the placenta such as the dopaminergic, the glucocorticoid and the brain-derived neurotrophic factor (BDNF) signalling pathway may also be involved in fetal neurodevelopment. A reduction in gene expression of the brain-derived neurotrophic factor (BDNF) pathway in human placental tissue and cord blood has been found to negatively affect neurodevelopment during childhood.[52] This pathway is involved in critical developmental processes, including embryo implantation, placental development, and fetal growth. Single nucleotide polymorphisms (SNPs) of genes of the serotonergic and dopaminergic pathways [53, 54] have been linked to neurological disorders, such as psychosis and schizophrenia.[55, 56] These pathways have also been characterized in relation to the placenta and neurological development. [57, 58] The concept of the placental regulation in brain development is relatively new and is in line with the observations of fetal programming and disease susceptibility later in life.[59] Epigenetic mechanisms, including promoter DNA methylation, are believed to play an essential role in this process. Indeed, recent studies have shown that epigenetic regulation through DNA methylation in the placenta is associated with infant neurobehavioural outcomes.[60, 61]

Groundbreaking nature and implications for twins

The intelligence score of twins and triplets is lower than the score of a singleton.[62] This difference in intelligence scores is partly attributed to differences in intrauterine growth.[62] However, to the best of our knowledge, the association between neurodevelopment and residential greenness, air pollution, traffic, noise and stress exposure *in utero* and during childhood has not been investigated in twins and triplets.

The originality lays in: I) studying the association between exposure to greenness, air pollution and stress and neurodevelopment using validated and objective neuropsycholocial tests and IQ together with high quality satellite data on greenness and conducting a follow-up design capable of linking exposures early in life in relation to the brain effects latter during the school period, II) assessing intermediate pathways such as inflammation in this association in a group of school age twins, III) the use of placental tissue as surrogate to study epigenetic targets of brain development and IV) the twin design to unravel genetic versus environmental effects.

Objectives and aim

Overall Aim: To study the association between neurodevelopment and residential surrounding greenness, air pollution and stress conditions, both social as maternal perceived stress, as well as biological stress (inflammation).

Specific aims:

1. To assess the relationship between home greenness (including traffic and land use) and cognitive development, behavioural symptoms and IQ
2. To investigate the underlying mechanisms, epigenetic and oxidative stress (of aim 1)
3. To assess the association between residential greenness and maternal stress
4. To evaluate the intermediate role of maternal stress in the relationship between residential air pollution and biological markers of oxidative stress/inflammation
5. To unravel nature versus nurture of cognition and behaviour
6. To assess on a prospective basis the relationship between residential air pollution, stress and its ultimate effect on cognitive development and behavioural symptoms in 4-year-old children (Future perspective)

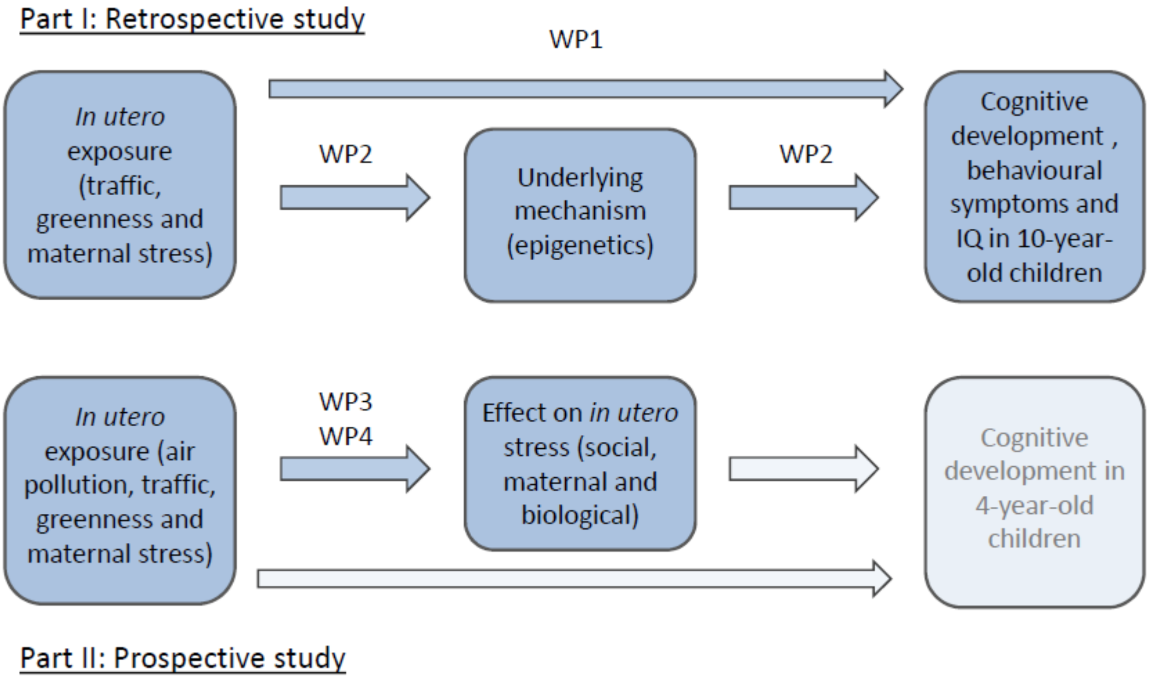

Figure: *Work flow of integrating early life exposures, mechanistic mediators and neurocognitive and neurobehavioural development. The time line of the different work packages (WP) is given on page 23 in the grant chart.* ***Overall research organisation:*** *Each WP is essential on the way towards better understanding of the cognitive development of twins in association with the environment. We study intelligence at age 7-15 and behaviour at age 6-17 in association with prenatal and postnatal exposure to residential surrounding greenness, traffic and maternal stress. To integrate the fetal origin of diseases in our study design, we will further study the determinants of cognition in association with prospective measures of maternal stress and air pollution exposure. To assess potential important biological markers, reflecting inflammatory conditions, telomere length and mitochondrial DNA content are measured in cord blood. The proper design of both prospective and retrospective analysis will in a cost efficient way lead to overall insight in early markers of cognitive function, intelligence as well as behaviour at schoolage. The environmental influence both including positive and negative insisters of neurocognition is a central issue throughout the concepts studied.*

METHODOLOGY

Part I: Cognitive development in 10-year old children and behavioural development in 8-year old childeren: retrospective study (WP1 and WP2 see table page 23)

Subject recruitment

The East Flanders Prospective Twin Survey (EFPTS) is a population based register of multiple births in the province of East-Flanders (Belgium) since 1964. The twins are ascertained at birth, placentation is recorded and placental biopsies were taken near the insertions of the umbilical cord.[63] Subjects in this study are recruited in two different sampling frames. First, all twin pairs (n = 5867) born between 1 September 1982 and 31 December 1991 were invited to complete the WISC-R (Wechsler Intelligence Scale for Children-Revised) test. The participation rate was 76% resulting in a final study population of 663 twin pairs with a mean age of 10.4 years old (between the ages of 7 and 15 years). In the second sampling frame, 1436 twin pairs were selected from the EFPTS. Their parents were asked to fill in the Child Behaviour Checklist (CBCL) and 760 parents (53%) participated in 1994-1996. Eight pairs were excluded. This resulted in a final study population of 752 pairs aged 6-17 years. Of the 752 pairs, 376 had also participated in the first part and also completed the WISC-R test. Besides the twins, also 52 triplet sets (140 children) performed the WISC-R test and the CBCL. The parents gave their written informed consent according to the local ethics committee guidelines.[64-66]

Exposure assessment

Residential addresses of the mothers at birth were geocoded. Distances to the nearest major road were determined using Geographic Information System (GIS) functions. All GIS analyses were conducted in ArcGIS 9.3. We collected information on two indicators of traffic at the residence: distance to major road, and traffic density. Traffic density in a specific radius (50 m, 100 m and 150 m) was equal to the length of each road in a buffer multiplied with the traffic count on each specific road. Airborne exhaust pollution from motor vehicles have high concentrations near roads and decline farther away in approximate Gaussian distribution.[67] Traffic densities were multiplied by a weight decreasing with distance, following a Gaussian curve. This model was originally developed and applied by Pearson.[67] Land use indicators such as semi-natural and forested -, agricultural -, residential and industrial area in a 5000 m radius from the residential address were estimated based on Corine land cover.

Maternal stress perception and assessment

Mothers will complete questionnaires retrospective about stress perception during pregnancy. The Perceived Stress Scale (PSS) is the most widely used psychological instrument for measuring the perception of stress. It is a measure of the degree to which situations in one’s life are appraised as stressful.

Neurodevelopment and behaviour

The neurodevelopment outcome was assessed by the Wechsler Intelligence Scale for Children-Revised (WISC-R). The WISC-R consists of six verbal and six performance subscales and has been validated for use in this population.[68] The verbal subscales are Information (INF), Similarities (SIM), Arithmetic (ARI), Vocabulary (VOC), Comprehension (COM) and Digit Span (DS). The performance subscales are Picture Completion (PC), Picture Arrangement (PA), Block Design (BD), Object Assembly (OA), Coding (COD) and Mazes (MAZ). The scores on the subscales are standardized for age and added up to Verbal (VIQ), Performance (PIQ) and Total Intelligence Quotients (TIQ). In this study, the total scores of the subscales and the TIQ score were analysed.

The neurobehavioural outcome was assessed by the Achenbach Child Behaviour Checklist (CBCL). The CBCL was developed by Achenbach (1991) to examine the extent to which children have behavioural and emotional problems as perceived by their parents. Although the CBCL allows for the calculation of separate scores corresponding to several behavioural dimensions based on exploratory factor analysis, in this study the total amount of psychopathology, as measured by the total problem score, was examined.

Underlying mechanism: retrospective study

Epigenetic mechanisms, including promoter DNA methylation, are believed to play an essential role in the process of placental regulation in brain development. We will investigate methylation of CpG sites within promoter regions of the most important genes involved in early neurodevelopment based on literature. Genes within several pathways will be studied since the serotenergic pathway, the dopaminergic, the glucocorticoid and the brain-derived neurotrophic factor (BDNF) signalling pathway may be involved in fetal neurodevelopment.

To investigate molecular effects of oxidative stress, biomarkers in processes associated with oxidative stress and inflammation will be measured. Postnatal measures of these biomarkers, telomere length and mitochondrial DNA content, will be applied in placental tissue.

<u>Part II: The intermediate role of stress exposure: prospective study (WP3-WP5 see table page 23)</u>

Subject recruitment

We will recruit mothers pregnant of twins in East-Flanders (Belgium). At birth besides placental tissue and cord blood, also maternal blood and hair will be collected. In addition, we asses stress during pregnancy and at time of birth.

Exposure assessment

Similar traffic and land-use variables will be measured as described in part I. In addition residential exposure to particulate air pollution will be determined. Regional background levels of PM_{10} for each mother's residential address will be estimated using a spatial temporal interpolation method (Kriging) that uses land cover data obtained from satellite images (Corine land cover data set) in combination

with monitoring stations (n = 34).[69, 70] This model provides interpolated daily PM_{10} values in 4 × 4 km grids from the Belgian telemetric air quality networks.

Maternal stress perception and assessment (Pilot study)

Maternal emotional and daily life stress reactivity will be assessed during one week in the third trimester of pregnancy. The Experience Sampling Method (ESM) will be used. This is a random timesampling self-assessment technique that has been shown to be feasible, valid, and reliable in general and patient populations.[71, 72] Subjects will receive a digital wristwatch that emits a signal ten times a day on five consecutive days, at unpredictable moments between 7:30 a.m. and 10:30 p.m. After each 'beep', subjects will complete ESM selfassessment forms concerning current context, thoughts, emotions, and psychotic experiences. Subjects will be instructed to complete their reports immediately after the beep, thus minimizing memory distortions.[73]

In addition, at time of birth mothers will receive a questionnaire; the Perceived Stress Scale (PSS). Cortisol, which is synthesized from cholesterol, is the main glucocorticoid in the zona fasciculata of human adrenal cortex and its secretion in response to biochemical stress contributes to the well-characterized suppression of the hypothalamic-pituitary-adrenal (HPA) axis on health and cognition events.[74-76] Since the vast majority of cortisol actions rely on binding to it cytosolic receptors, only the small fraction of unbound, free cortisol is revealed to be biologically active. It comes out of the mitochondrion and moves out of the cell into the extracellular space and into the bloodstream. Due to its low molecular weight and lipophilic nature, unbound cortisol enters cells by passive diffusion that makes it feasible to measure the free cortisol in many body tissues.[77] In this study, maternal cortisol levels during pregnancy will be measured in hair. Measurement of cortisol concentrations in hair is a non-invasive method and a better tool for evaluating chronic stress compared to salivary cortisol.[78] Since hair grows approximately one cm per month, we will collect 9 cm of maternal hair at time of delivery to reflect the 9 months of pregnancy.[79]

Inflammation & oxidative stress

Oxidative and nitrosative stress is postulated to be one of the mechanisms by which particulate matter (PM) exerts its effects.[80] Organic chemical components onto the particle surface play an important role in the production of reactive oxygen species (ROS). Mitochondrial DNA (mtDNA) molecules are highly susceptible to oxidative stress. Accumulation of mtDNA mutations leads to alterations of mitochondrial biogenesis and function that might result in decrease of mtDNA content within cells and implies a role of mtDNA content as a potential biomarkers in processes associated with oxidative stress and inflammation. In addition, telomere shortening is a marker of exposure to life time oxidative stress. Postnatal measures of these inflammation biomarkers will be applied in cord blood.

STATISTICAL ANALYSIS PLAN

Mixed modelling will be performed to assess the association between environmental factors and outcomes. The twins will be analyzed as individuals in a multilevel regression analysis by adding a random intercept to the model to adjust for the relatedness between twin members. Covariables and potential confounders will be identified, including newborn's sex, age, parental education, neighbourhood socioeconomic status. We will use a prospective observational design. First, we will investigate the association between exposure (environmental factors) and outcome (cognitive development, behavioural symptoms and IQ), between exposure and mediators (epigenetic and oxidative stress) and between mediators and outcome. To investigate the intermediate role of oxidative stress in the relationship between the residential environment and outcome (cognitive development, behavioural symptoms and IQ), we will use mediation analysis. The mediation analysis will be performed if there is a significant association between the outcome and the exposure (environmental factors), a significant association between the exposure and the mediator (epigenetic and oxidative stress), and a significant association between the outcome and the mediator. We will use a SAS macro developed by Valeri and VanderWeele. In this macro, the direct effect (DE), indirect effect (IE) and total effect (TE) are determined. The DE represents the effect of exposure on the outcome after controlling for the mediator whereas the IE is the effect of exposure operating through the mediator. The proportion of mediation is calculated as the ratio of IE to TE.

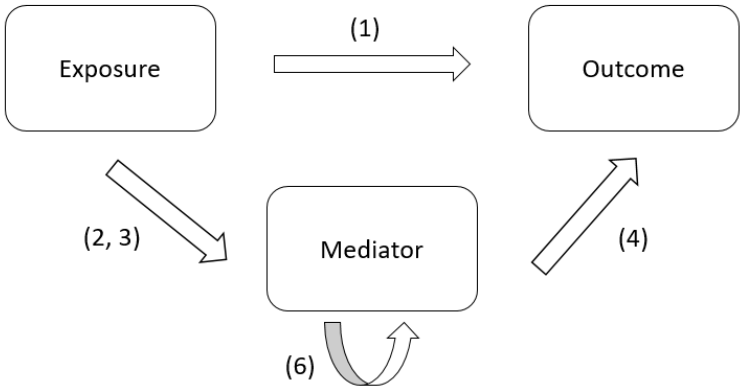

Finally, twin research provides the opportunity to gain insight into the relative importance of genes and environment or nature vs nurture aspects. The classic twin study can unravel nature vs nurture by comparing the intrapair similarity of monozygotic twins with dizygotic twins. This can be investigated using structural equation modelling with Open Mx software. Based on the classic ACE model, the variance will be decomposed in additive genetic (A), common environmental (C), and unique environmental (E) effects. This is the classic ACE model. The model makes use of the fact that MZ twins are genetically identical, whereas DZ twins share half of their genes. Furthermore, the model assumes that MZ and DZ twins share their common environment to the same extent. The result of this assumption is that a greater within-pair similarity in MZ twins than in DZ twins reflects genetic influences. Using the twin design, the relative influence of genetic and environmental factors on birth outcomes can be estimated (aim 5).

FUTURE PERSPECTIVES

We designed the study in a specific way that after 4 years we not only provided a finished project but also as a stepping stone to new projects. The twins recruited in part II can be invited to a follow-up study at age 4 year. Measurements of neurodevelopment and micro-circulation at this follow-up can be studied in relation to previous determined parameters; air pollution and stress exposure, molecular markers of inflammation/oxidative stress.

Micro-circulation

Changes in the microcirculation can be explored non-invasively by studying retinal blood vessels that are visualized in fundus images,[81, 82] the micro-circulation might represents brain functions. The retinal blood vessels have anatomical and physiological features that are comparable with the coronary circulation. Pathologies of the retinal blood vessels parallel changes in the coronary micro- and macrocirculation.[83-85] Nowadays, the retinal vessel caliber is regarded as an independent risk factor for cardiovascular diseases with arterial narrowing acting as a marker for arteriolar damage and predicting hypertension. Venular widening is associated with inflammation, endothelial dysfunction and markers of atherosclerosis.[82, 85, 86]

Retinal photography provides a noninvasive, in vivo, method for characterizing the human microvasculature since retinal vessels are 60–300 mm in diameter. Several studies have found that narrower arteriolar diameters and wider venular diameters, as measured by retinal photography, are each associated with increased risk of myocardial infarction, stroke, and cardiummovascular mortality, independent of other traditional risk factors.[87] For each participant, the optic disc and macula of both eyes is photographed in a darkened room using a 45° 6.3-megapixel digital nonmydriatic camera (Canon), and methods described in detail by Klein and colleagues.[88] Retinal vessel diameters are measured using a computer-based program and sarized as central retinal arteriolar and venular equivalents (CRAE and CRVE, respectively).[86, 87, 89]

Neurodevelopment

For assessing working memory and attention tasks, it is possible to uase CANTAB (Cambridge Cognition Ltd). Motor Screening Test (MOT), Big/Little Circle Test (BLC), Spatial Span Test (SSP), Paired Associates Learning Test (PAL) and Delayed Matching to Sample Test (DMS) can be applied in order to investigate induction, attention, executive function and visual memory, respectively. These functions specifically developed during school childhood period. At the same time, these cognitive functions have been shown to be very sensitive to environmental hazards.[90] Strengths and Difficulties Questionnaire (SDQ) can be filled in by parents in order to characterise behavioural traits including hyperactivity/inattention, peer relationship, emotion, and conduct.

This chart represents the timing of the different Work Packages (WPs) during this 2-year project and a possible extension with 2 additional years.

	Year 1		Year 2		Year 3		Year 4	
**Part I: Retrospective study**								
**WP1 - Neurodevelopment at school age**								
WP1.1. Exposure assessment	■							
WP1.2. Twin design		■						
WP1.3. Statistical analysis		■						
WP1.4. Writing Scientific publications			■					
**WP2 - Underlying mechanism**								
WP2.1. Epigenetics – DNA methylation	■	■						
WP2.2. Biomarkers of oxidative stress		■	■					
WP2.3. Twin design			■					
WP2.4. Statistical analysis			■				■	
WP2.5. Writing Scientific publications				■				■
**Part II: Prospective study**								
**WP3 - Study population and data collection**								
WP3.1. Recruitment	■	■	■	■	■	■	■	
WP3.2. Stress measurement	■	■	■	■	■	■	■	■
WP3.3. Blood/tissue collection	■	■	■	■	■	■	■	■
WP3.4. Exposure assessment				■			■	
**WP4 - Molecular and biochemical measurements**								
WP4.1. DNA extraction			■	■	■	■		
WP4.2. Telomere length					■	■		
WP4.3. Mitochondrial DNA content					■	■		
WP4.5. Cortisol					■	■		
**WP5 - Statistics and reporting**								
WP5.1. Twin design						■		
WP5.2. Statistical analysis							■	
WP5.3. Writing scientific publications								■

REFERENCES

1. Amoly E, Dadvand P, Forns J*, et al.* Green and blue spaces and behavioral development in Barcelona schoolchildren: the BREATHE project. *Environ Health Perspect* 2014;122(12):1351-8.
2. Markevych I, Tiesler CM, Fuertes E*, et al.* Access to urban green spaces and behavioural problems in children: Results from the GINIplus and LISAplus studies. *Environ Int* 2014;71:29-35.
3. Wells. At home with nature; Effects of "Greenness" on Children's Cognitive Functioning. *Environment and Behavior* 2000;32(6):775-95.
4. Taylor AF, Kuo FE. Children with attention deficits concentrate better after walk in the park. *J Atten Disord* 2009;12(5):402-9.
5. Kuo FE, Taylor AF. A potential natural treatment for attention-deficit/hyperactivity disorder: evidence from a national study. *Am J Public Health* 2004;94(9):1580-6.
6. van den Berg AE, Maas J, Verheij RA, Groenewegen PP. Green space as a buffer between stressful life events and health. *Soc Sci Med* 2010;70(8):1203-10.
7. Wu CD, McNeely E, Cedeno-Laurent JG*, et al.* Linking student performance in Massachusetts elementary schools with the "greenness" of school surroundings using remote sensing. *PLoS One* 2014;9(10):e108548.
8. Miller KA, Siscovick DS, Sheppard L*, et al.* Long-term exposure to air pollution and incidence of cardiovascular events in women. *N Engl J Med* 2007;356(5):447-58.
9. Nawrot TS, Nemmar A, Nemery B. Air pollution: To the heart of the matter. *Eur Heart J* 2006;27(19):2269-71.
10. Calderon-Garciduenas L, Solt AC, Henriquez-Roldan C*, et al.* Long-term air pollution exposure is associated with neuroinflammation, an altered innate immune response, disruption of the blood-brain barrier, ultrafine particulate deposition, and accumulation of amyloid beta-42 and alpha-synuclein in children and young adults. *Toxicologic Pathology* 2008;36(2):289-310.
11. Calderon-Garciduenas L, Franco-Lira M, Henriquez-Roldan C*, et al.* Urban air pollution: influences on olfactory function and pathology in exposed children and young adults. *Experimental and Toxicologic Pathology* 2010;62(1):91-102.
12. Oberdorster G, Sharp Z, Atudorei V*, et al.* Translocation of inhaled ultrafine particles to the brain. *Inhalation Toxicology* 2004;16(6-7):437-45.
13. Elder A, Gelein R, Silva V*, et al.* Translocation of inhaled ultrafine manganese oxide particles to the central nervous system. *Environmental Health Perspectives* 2006;114(8):1172-78.
14. Dorman DC, McManus BE, Parkinson CU, Manuel CA, McElveen AM, Everitt JI. Nasal toxicity of manganese sulfate and manganese phosphate in young male rats following subchronic (13-week) inhalation exposure. *Inhalation Toxicology* 2004;16(6-7):481-8.
15. Tjalve H, Henriksson J, Tallkvist J, Larsson BS, Lindquist NG. Uptake of manganese and cadmium from the nasal mucosa into the central nervous system via olfactory pathways in rats. *Pharmacology and Toxicology* 1996;79(6):347-56.
16. Oberdorster G, Sharp Z, Atudorei V*, et al.* Extrapulmonary translocation of ultrafine carbon particles following whole-body inhalation exposure of rats. *Journal of Toxicology and Environmental Health Part A* 2002;65(20):1531-43.
17. Furuyama A, Kanno S, Kobayashi T, Hirano S. Extrapulmonary translocation of intratracheally instilled fine and ultrafine particles via direct and alveolar macrophage-associated routes. *Archives of Toxicology* 2009;83(5):429-37.
18. Refsnes M, Hetland RB, Ovrevik J, Sundfor I, Schwarze PE, Lag M. Different particle determinants induce apoptosis and cytokine release in primary alveolar macrophage cultures. *Particle and Fibre Toxicology* 2006;3:10.
19. Van Eeden SF, Tan WC, Suwa T*, et al.* Cytokines involved in the systemic inflammatory response induced by exposure to particulate matter air pollutants (PM(10)). *American Journal of Respiratory and Critical Care Medicine* 2001;164(5):826-30.

20. Sawyer K, Mundandhara S, Ghio AJ, Madden MC. The effects of ambient particulate matter on human alveolar macrophage oxidative and inflammatory responses. *Journal of Toxicology and Environmental Health Part A* 2010;73(1):41-57.
21. Huang YC, Li Z, Harder SD, Soukup JM. Apoptotic and inflammatory effects induced by different particles in human alveolar macrophages. *Inhalation Toxicology* 2004;16(14):863-78.
22. Devlin RB, McKinnon KP, Noah T, Becker S, Koren HS. Ozone-induced release of cytokines and fibronectin by alveolar macrophages and airway epithelial cells. *American Journal of Physiology* 1994;266(6 Pt 1):L612-9.
23. Kido T, Tamagawa E, Bai N*, et al.* Particulate matter induces translocation of IL-6 from the lung to the systemic circulation. *American Journal of Respiratory Cell and Molecular Biology* 2011;44(2):197-204.
24. Tan WC, Qiu D, Liam BL*, et al.* The human bone marrow response to acute air pollution caused by forest fires. *American Journal of Respiratory and Critical Care Medicine* 2000;161(4 Pt 1):1213-7.
25. Hogg JC, Van Eeden S. Pulmonary and systemic response to atmospheric pollution. *Respirology* 2009;14(3):336-46.
26. Mukae H, Hogg JC, English D, Vincent R, Van Eeden SF. Phagocytosis of particulate air pollutants by human alveolar macrophages stimulates the bone marrow. *American Journal of Physiology: Lung Cellular and Molecular Physiology* 2000;279(5):L924-31.
27. Perera FP, Rauh V, Whyatt RM*, et al.* Effect of prenatal exposure to airborne polycyclic aromatic hydrocarbons on neurodevelopment in the first 3 years of life among inner-city children. *Environ Health Perspect* 2006;114(8):1287-92.
28. Perera FP, Rauh V, Tsai WY*, et al.* Effects of transplacental exposure to environmental pollutants on birth outcomes in a multiethnic population. *Environ Health Perspect* 2003;111(2):201-5.
29. Edwards SC, Jedrychowski W, Butscher M*, et al.* Prenatal exposure to airborne polycyclic aromatic hydrocarbons and children's intelligence at 5 years of age in a prospective cohort study in Poland. *Environmental Health Perspectives* 2010;118(9):1326-31.
30. Chiu YH, Bellinger DC, Coull BA*, et al.* Associations between traffic-related black carbon exposure and attention in a prospective birth cohort of urban children. *Environ Health Perspect* 2013;121(7):859-64.
31. Calderon-Garciduenas L, Mora-Tiscareno A, Ontiveros E*, et al.* Air pollution, cognitive deficits and brain abnormalities: a pilot study with children and dogs. *Brain and Cognition* 2008;68(2):117-27.
32. Calderon-Garciduenas L, Engle R, Mora-Tiscareno A*, et al.* Exposure to severe urban air pollution influences cognitive outcomes, brain volume and systemic inflammation in clinically healthy children. *Brain and Cognition* 2011;77(3):345-55.
33. Beyer KM, Kaltenbach A, Szabo A, Bogar S, Nieto FJ, Malecki KM. Exposure to neighborhood green space and mental health: evidence from the survey of the health of Wisconsin. *Int J Environ Res Public Health* 2014;11(3):3453-72.
34. Ward Thompson C, Roe J, Aspinall P, Mitchell R, Clow A, Miller D. More green space is linked to less stress in deprived communities: Evidence from salivary cortisol patterns. *Landscape and Urban Planning* 2012;105(3):221-29.
35. Niederhofer H, Reiter A. Prenatal maternal stress, prenatal fetal movements and perinatal temperament factors influence behavior and school marks at the age of 6 years. *Fetal Diagn Ther* 2004;19(2):160-2.
36. Huizink AC, Robles de Medina PG, Mulder EJ, Visser GH, Buitelaar JK. Stress during pregnancy is associated with developmental outcome in infancy. *J Child Psychol Psychiatry* 2003;44(6):810-8.
37. Talge NM, Neal C, Glover V. Antenatal maternal stress and long-term effects on child neurodevelopment: how and why? *J Child Psychol Psychiatry* 2007;48(3-4):245-61.
38. Charil A, Laplante DP, Vaillancourt C, King S. Prenatal stress and brain development. *Brain Res Rev* 2010;65(1):56-79.

39. Seckl JR, Holmes MC. Mechanisms of disease: glucocorticoids, their placental metabolism and fetal 'programming' of adult pathophysiology. *Nat Clin Pract Endocrinol Metab* 2007;3(6):479-88.
40. Avishai-Eliner S, Brunson KL, Sandman CA, Baram TZ. Stressed-out, or in (utero)? *Trends Neurosci* 2002;25(10):518-24.
41. Coe CL, Kramer M, Czeh B*, et al.* Prenatal stress diminishes neurogenesis in the dentate gyrus of juvenile rhesus monkeys. *Biol Psychiatry* 2003;54(10):1025-34.
42. Schmitz C, Rhodes ME, Bludau M*, et al.* Depression: reduced number of granule cells in the hippocampus of female, but not male, rats due to prenatal restraint stress. *Mol Psychiatry* 2002;7(7):810-3.
43. Sjostrom K, Valentin L, Thelin T, Marsal K. Maternal anxiety in late pregnancy and fetal hemodynamics. *Eur J Obstet Gynecol Reprod Biol* 1997;74(2):149-55.
44. von Zglinicki T. Role of oxidative stress in telomere length regulation and replicative senescence. *AnnNYAcadSci* 2000;908:99-110.
45. Reliene R, Hlavacova A, Mahadevan B, Baird WM, Schiestl RH. Diesel exhaust particles cause increased levels of DNA deletions after transplacental exposure in mice. *MutatRes* 2005;570(2):245-52.
46. Bijnens E, Zeegers MP, Gielen M*, et al.* Lower placental telomere length may be attributed to maternal residential traffic exposure; a twin study. *Environ Int* 2015;79:1-7.
47. Janssen BG, Munters E, Pieters N*, et al.* Placental mitochondrial DNA content and particulate air pollution during in utero life. *Environ Health Perspect* 2012;120(9):1346-52.
48. Zeltser LM, Leibel RL. Roles of the placenta in fetal brain development. *Proc Natl Acad Sci U S A* 2011;108(38):15667-8.
49. Muotri AR, Gage FH. Generation of neuronal variability and complexity. *Nature* 2006;441(7097):1087-93.
50. Broad KD, Keverne EB. Placental protection of the fetal brain during short-term food deprivation. *Proc Natl Acad Sci U S A* 2011;108(37):15237-41.
51. Bonnin A, Levitt P. Fetal, maternal, and placental sources of serotonin and new implications for developmental programming of the brain. *Neuroscience* 2011;197:1-7.
52. Dhobale M, Mehendale S, Pisal H, D'Souza V, Joshi S. Association of brain-derived neurotrophic factor and tyrosine kinase B receptor in pregnancy. *Neuroscience* 2012;216:31-7.
53. Spencer GE, Klumperman J, Syed NI. Neurotransmitters and neurodevelopment. Role of dopamine in neurite outgrowth, target selection and specific synapse formation. *Perspect Dev Neurobiol* 1998;5(4):451-67.
54. Homberg JR, Molteni R, Calabrese F, Riva MA. The serotonin-BDNF duo: developmental implications for the vulnerability to psychopathology. *Neurosci Biobehav Rev* 2014;43:35-47.
55. Melkersson K, Hulting AL. Serotonin receptor 2A gene polymorphisms and schizophrenia: association with family history, diagnostic subtype and height in patients. *Neuro Endocrinol Lett* 2009;30(3):343-51.
56. Andreou D, Soderman E, Axelsson T*, et al.* Polymorphisms in genes implicated in dopamine, serotonin and noradrenalin metabolism suggest association with cerebrospinal fluid monoamine metabolite concentrations in psychosis. *Behav Brain Funct* 2014;10:26.
57. Yang CJ, Tan HP, Du YJ. The developmental disruptions of serotonin signaling may involved in autism during early brain development. *Neuroscience* 2014;267:1-10.
58. Petit A, Vaillancourt C, Bellabarba D, Lehoux JG, Gallo-Payet N, Belisle S. Presence of D2-dopamine receptors in human term placenta. *J Recept Res* 1990;10(3-4):205-15.
59. Barker DJ. The fetal and infant origins of adult disease. *Bmj* 1990;301(6761):1111.
60. Paquette AG, Lesseur C, Armstrong DA*, et al.* Placental HTR2A methylation is associated with infant neurobehavioral outcomes. *Epigenetics* 2013;8(8):796-801.
61. Lesseur C, Armstrong DA, Murphy MA*, et al.* Sex-specific associations between placental leptin promoter DNA methylation and infant neurobehavior. *Psychoneuroendocrinology* 2014;40:1-9.

62. Eriksen W, Sundet JM, Tambs K. Twin-singleton differences in intelligence: a register-based birth cohort study of Norwegian males. *Twin Res Hum Genet* 2012;15(5):649-55.
63. Derom C, Thiery E, Peeters H, Vlietinck R, Defoort P, Frijns JP. The East Flanders Prospective Twin Survey (EFPTS): an actual perception. *Twin Res Hum Genet* 2013;16(1):58-63.
64. Jacobs N, Rijsdijk F, Derom C*, et al.* Child psychopathology and lower cognitive ability: a general population twin study of the causes of association. *Mol Psychiatry* 2002;7(4):368-74.
65. Rizzi TS, van der Sluis S, Derom C*, et al.* Genetic Variance in Combination with Fatty Acid Intake Might Alter Composition of the Fatty Acids in Brain. *PLoS One* 2013;8(6):e68000.
66. Antoniou EE, Fowler T, Thiery E*, et al.* Intrauterine environment and cognitive development in young twins. *J Dev Orig Health Dis* 2013;4(6):513-21.
67. Pearson RL, Wachtel H, Ebi KL. Distance-weighted traffic density in proximity to a home is a risk factor for leukemia and other childhood cancers. *J Air Waste Manag Assoc* 2000;50(2):175-80.
68. Wechsler D. *Wechsler Intelligence Scale for Children-R (Dutch version)*: Swets and Zeitlinger B. V., Lisse, the Netherlands, 1986.
69. Janssen S, Dumont G, Fierens F, Mensink C. Spatial interpolation of air pollution measurements using CORINE land cover data. *Atmospheric Environment* 2008;42(20):4884-903.
70. Jacobs L, Emmerechts J, Mathieu C*, et al.* Air pollution related prothrombotic changes in persons with diabetes. *EnvironHealth Perspect* 2010;118(2):191-96.
71. Csikszentmihalyi M, Larson R. Validity and reliability of the Experience-Sampling Method. *J Nerv Ment Dis* 1987;175(9):526-36.
72. Myin-Germeys I, Oorschot M, Collip D, Lataster J, Delespaul P, van Os J. Experience sampling research in psychopathology: opening the black box of daily life. *Psychol Med* 2009;39(9):1533-47.
73. Collip D, Wigman JT, Myin-Germeys I*, et al.* From epidemiology to daily life: linking daily life stress reactivity to persistence of psychotic experiences in a longitudinal general population study. *PLoS One* 2013;8(4):e62688.
74. Staab CA, Maser E. 11beta-Hydroxysteroid dehydrogenase type 1 is an important regulator at the interface of obesity and inflammation. *J Steroid Biochem Mol Biol* 2010;119(1-2):56-72.
75. Oswald LM, Zandi P, Nestadt G, Potash JB, Kalaydjian AE, Wand GS. Relationship between cortisol responses to stress and personality. *Neuropsychopharmacology* 2006;31(7):1583-91.
76. Juster RP, McEwen BS, Lupien SJ. Allostatic load biomarkers of chronic stress and impact on health and cognition. *Neurosci Biobehav Rev* 2010;35(1):2-16.
77. Miller WL. Steroidogenic enzymes. *Endocr Dev* 2008;13:1-18.
78. Iglesias S, Jacobsen D, Gonzalez D*, et al.* Hair cortisol: A new tool for evaluating stress in programs of stress management. *Life Sci* 2015;141:188-92.
79. Karlen J, Frostell A, Theodorsson E, Faresjo T, Ludvigsson J. Maternal influence on child HPA axis: a prospective study of cortisol levels in hair. *Pediatrics* 2013;132(5):e1333-40.
80. Rossner P, Jr., Svecova V, Milcova A*, et al.* Oxidative and nitrosative stress markers in bus drivers. *Mutat Res* 2007;617(1-2):23-32.
81. Wong TY, Klein R, Couper DJ*, et al.* Retinal microvascular abnormalities and incident stroke: the Atherosclerosis Risk in Communities Study. *Lancet* 2001;358(9288):1134-40.
82. Wong T, Mitchell P. The eye in hypertension (vol 369, pg 426, 2007). *Lancet* 2007;369(9579):2078-78.
83. Tso MO, Jampol LM. Pathophysiology of hypertensive retinopathy. *Ophthalmology* 1982;89(10):1132-45.
84. Tedeschi-Reiner E, Strozzi M, Skoric B, Reiner Z. Relation of atherosclerotic changes in retinal arteries to the extent of coronary artery disease. *Am J Cardiol* 2005;96(8):1107-9.
85. Nguyen TT, Wong TY. Retinal vascular manifestations of metabolic disorders. *Trends Endocrinol Metab* 2006;17(7):262-8.
86. Wong TY, Klein R, Sharrett AR*, et al.* Retinal arteriolar diameter and risk for hypertension. *Annals of Internal Medicine* 2004;140(4):248-55.

87. Adar SD, Klein R, Klein BE*, et al.* Air Pollution and the microvasculature: a cross-sectional assessment of in vivo retinal images in the population-based multi-ethnic study of atherosclerosis (MESA). *PLoS Med* 2010;7(11):e1000372.
88. Klein R, Klein BE, Knudtson MD*, et al.* Prevalence of age-related macular degeneration in 4 racial/ethnic groups in the multi-ethnic study of atherosclerosis. *Ophthalmology* 2006;113(3):373-80.
89. Knudtson MD, Lee KE, Hubbard LD, Wong TY, Klein R, Klein BE. Revised formulas for summarizing retinal vessel diameters. *Curr Eye Res* 2003;27(3):143-9.
90. Sunyer J, Esnaola M, Alvarez-Pedrerol M*, et al.* Association between Traffic-Related Air Pollution in Schools and Cognitive Development in Primary School Children: A Prospective Cohort Study. *PLoS Med* 2015;12(3):e1001792.

CURRICULUM VITAE

Esmée Bijnens was born in March 1988 in Genk, Belgium. She studied Biology at Hasselt University. In 2009 she graduated at Hasselt University and the same year she started the master Cel- en Systeembiologie at Antwerp University. This gave her the opportunity to specialize in molecular biology. During her masterthesis she studied post-transcriptional modifications in histones in *Brassica napus*. She graduated in 2011 with distinction at Antwerp University. Subsequently she started a Joint PhD at the Centre for Environmental Sciences at Hasselt University (Prof. Dr. Tim Nawrot) and the Department of Complex Genetics at Maastricht University (Prof. Dr. Maurice Zeegers). In 2012, she followed a post-academic course "Modelling of Environmental Exposures Using GIS" at Imperial College, London. In 2014, she joined the International Workshop on Statistical Genetic Methods for Human Complex Traits at the University of Colorado.

During her PhD she investigated the effect of traffic related exposure in early-life in twins. Important traffic parameters can be calculated via a Geographic Information System (GIS). She applied this geo-information system in on one of the world largest longitudinal prospective twin cohort. The East Flanders Prospective Twin Survey (EFPTS), a Belgian population based register which started in 1964, offers the opportunity to follow up twins from birth to adulthood. Environmental influences on the intrauterine environment may not only effect fetal growth but also may predispose offspring to increased risk of clinical outcomes later in life. Her research shows that prenatal exposure to air pollution and traffic was respectively associated with a suboptimal fetal growth and a change in placental telomere length, a biomarker of ageing. Besides these outcomes at birth, she noted that exposure to traffic early in life was associated with shorter telomere length in young adulthood and that surrounding greenness in early-life is associated with a lower blood pressure in adulthood.

LIST OF PUBLICATIONS

Articles in peer-reviewed journals

1. **Bijnens EM,** Derom C, Gielen M, Winckelmans E, Fierens F, Vlietinck R, Zeegers MP, Nawrot TS. Small for gestational age and exposure to particulate air pollution in the early-life environment of twins. ***Environ Res.*** 2016; 148: 39-45.

2. Saenen ND, Plusquin M, **Bijnens E,** Janssen BG, Gyselaers W, Cox B, Fierens F, Molenberghs G, Penders J, Vrijens K, De Boever P and Nawrot TS. In Utero Fine Particulate Air Pollution and Placental Expression of Genes in the Brain-Derived Neurotrophic Factor Signaling Pathway: An ENVIRONAGE Birth Cohort Study. ***Environ Health Perspect.*** 2015; 123: 834-40.

3. **Bijnens E,** Zeegers MP, Gielen M, Kicinski M, Hageman Gj, Pachen D, Derom C, Vlietinck R and Nawrot TS. Lower placental telomere length may be attributed to maternal residential traffic exposure; a twin stud. ***Environ int.*** 2015; 79: 1-7.

4. Kicinski M, Vermeir G, Van Larebeke N, Den Hond E, Schoeters G, Bruckers L, Sioen I, **Bijnens E,** Roels HA, Baeyens W, Viaene MK and Nawrot TS. Neurobehavioral performance in adolescents is inversely associated with traffic exposure. ***Environ int*.** 2015; 75:136-43.

5. Goeminne PC, **Bijnens E,** Nemery B, Nawrot TS and Bupont L. Impact of traffic related air pollution indicators of non-cystic fibrosis bronchiectasis mortality: a cohort analysis. ***Respir Res.*** 2014; 15:108.

6. **Bijnens E,** Pieter N, Dewitte H, Cox B, Janssen BG, Saenen N, Dons E, Zeegers MP, Int Panis L and Nawrot TS. Host and environmental predictors of exhaled breath temperature in the elderly. ***BMC Public Health.*** 2013; 13:1226.

Conference material

1. **Bijnens E,** Zeegers MP, Loos R, Gielen M, Vlietinck R, Derom C and Nawrot TS. Blood pressure in young adulthood and residential exposure of traffic and greenness in the early-life environment of twins. ISEE Early *Career Researchers Conference on Environmental Epidemiology; Utrecht; The Netherlands;* 2015.

2. **Bijnens E,** Nawrot TS, Derom C, Janssen BG, Vrijens K, Vlietinck R, Gielen M and Zeegers MP, Maurice P. Maternal residential greenness and traffic indicators in association with alterations in placental mitochondrial DNA content in twins ISEE Early *Career Researchers Conference on Environmental Epidemiology; Utrecht; The Netherlands;* 2015.

3. **Bijnens E,** Derom C, Gielen M, Winckelmans E, Fierens F, Vlietinck R, Zeegers MP and Nawrot TS. Fetal growth restriction and exposure to particulate air pollution in the early-life environment of twins. *Healthy Living Conference; Maastricht; The Netherlands;* 2015.

4. **Bijnens E,** Zeegers MP, Gielen M, Kicinski M, Hageman Gj, Pachen D, Derom C, Vlietinck R and Nawrot TS. Lower placental telomere length may be attributed to maternal residential traffic exposure; a twin study. *Healthy Living Conference; Maastricht; The Netherlands;* 2015.

5. **Bijnens E,** Derom C, Gielen M, Winckelmans E, Fierens F, Vlietinck R, Zeegers MP and Nawrot TS. Strong associations between particulate air pollution and fetal growth in twins. *World Congress on Twin Pregnancy and International Society of Twin Studies (ISTS); 2014; Budapest, Hungary,* 2014.

6. **Bijnens E,** Zeegers MP, Gielen M, Kicinski M, Hageman Gj, Pachen D, Derom C, Vlietinck R and Nawrot TS. Lower placental telomere length may be attributed to maternal residential traffic exposure; a twin study. *ISTS Twins 2014; Budapest, Hungary,* 2014.

7. **Bijnens E,** Zeegers MP, Gielen M, Kicinski M, Hageman Gj, Pachen D, Derom C, Vlietinck R and Nawrot TS. Lower placental telomere length may be attributed to maternal residential traffic exposure; a twin study. ISEE *Young Researchers Conference on Environmental Epidemiology; Barcelona; Spain;* 2014.

8. **Bijnens E,** Pieter N, Dewitte H, Cox B, Janssen BG, Saenen N, Dons E, Zeegers MP, Int Panis L and Nawrot TS. Host and environmental predictors of exhaled breath temperature in the elderly. *Environment and Health: Bridging South, North, East and West; Basel, Switzerland;* 2013.

KEY PUBLICATIONS OF PROMOTORS OF LAST 5 YEARS

-several of these references were granted by the Marguerite-Marie Delacroix Foundation

Tim Nawrot

Saenen ND, Plusquin M, Bijnens E, Janssen BG, Gyselaers W, Cox B, Fierens F, Molenberghs G, Penders J, Vrijens K, De Boever P and Nawrot TS. *In Utero Fine Particulate air pollution and placental expression of genes in the Brain-Derived Neurotrophic Factor signalling pathway: An ENVIRONAGE Birth Cohort Study.* ***Environ Health Persp*** *2015; 123(8):* 834-840.

Cox B, Martens E, Nemery B, Vangronsveld J and Nawrot TS. *Impact of a stepwise introduction of smoke-free legislation on the rate of preterm births: analysis of routinely collected birth data.* ***BMJ*** 2013; 346:f441.

Nawrot TS, Perez L, Kunzli N, Munters E and Nemery B. *Public health importance of triggers of myocardial infarction: a compative risk assessment.* ***Lancet*** 2011; 377 (9767): 732-40.

Kicinski M, Vermeir G, Van Larebeke N, Den Hond E, Schoeters G, Bruckers L, Sioen I, Bijnens E, Roels HA, Baeyens W, Viaene MK and Nawrot TS. *Neurobehavioral performance in adolescents in inversely associated with traffic exposure.* ***Environ int*** 2015; 75: 136-43.

Bijnens E, Zeegers MP, Gielen M, Kicinski M, Hagenman GJ, Pachen D, Derom C, Vlietinck R and Nawrot TS. *Lower placental telomere length may be attributed to maternal residential traffic exposure; a twin study.* ***Environ int*** 2015; 79: 1-7.

Janssen BG, Munters E, Pieters N, Smeets K, Cox B, Cuypers A, Fierens F, Penders J, Vangrondsveld J, Gyselaers W and Nawrot TS. *Placental mitochondrial DNA content and particulate air pollution during in utero life.* ***Environ Health Persp*** 2012; 120(9): 1346-52.

Catherine Derom

Derom C, Thiery E, Peeters H, Vlietinck R, Defoort P and Frijns JP. The East Flanders Prospective Twin Survey (EFPTS). An Actual Perception. ***Twin Res Hum Genet*** 2013; 16(1):58-63.

Wichers M, Maes HH, Jacobs N, Derom C, Thiery E and Kendler KS. Disentangling the causal inter-relationship between negative life events and depressive symptoms in women: a longitudinal twin study. ***Psychol Med*** 2012; 42(9):1801-14.

Rizzi TS, van der Sluis S, Derom C, Thiery E, van Kesteren RE, Jacobs N, Van Gestel S, Vlietinck R, Verhage M, Heutink P and Posthuma D. FADS2 Genetic Variance in Combination with Fatty Acid Intake Might Alter Composition of the Fatty Acids in Brain. ***PLoS One*** 2013; 8(6):e68000.

Collip D, Wigman JT, Myin-Germeys I, Jacobs N, Derom C, Thiery E, Wichers M and van Os J. From epidemiology to daily life: linking daily life stress reactivity to persistence of psychotic experiences in a longitudinal general population study. ***PLoS One*** 2013; 8(4):e62688.

Bijnens EM, Derom C, Gielen M, Winckelmans E, Fierens F, Vlietinck R, Zeegers MP, Nawrot TS. Small for gestational age and exposure to particulate air pollution in the early-life environment of twins. ***Environ Res.*** 2016; 148: 39-45.

Evert Thiery

Derom C, Thiery E, Peeters H, Vlietinck R, Defoort P and Frijns JP. The East Flanders Prospective Twin Survey (EFPTS). An Actual Perception. ***Twin Res Hum Genet*** 2013; 16(1):58-63.

Collip D, Myin-Germeys I, Wichers M, Jacobs N, Derom C, Thiery E, Lataster T, Simons C, Delespaul P, Marcelis M, van Os J and van Winkel R. FKBP5 as a possible moderator of the psychosis-inducing effects of childhood trauma. ***Br J Psychiatry*** 2013; 202(4): 261-8.

Wigman JT, van Os J, Thiery E, Derom C, Collip D, Jacobs N and Wichers M. Psychiatric diagnosis revisited: towards a system of staging and profiling combining nomothetic and idiographic parameters of momentary mental states. ***PLoS One*** 2013; 8(3):e59559.

Antoniou E, Fowler T, Thiery E, Southwood TR, van Gestel S, Jacobs N, Vlietinck R, van Os J, Rijsdijk FV, Derom C and Zeegers MP. Intrauterine environment and cognitive development in young twins. ***J Dev Orig Health Dis*** 2013; 4(6): 513–521.

van Winkel M, Peeters F, van Winkel R, Kenis G, Collip D, Geschwind N, Jacobs N, Derom C, Thiery E, van Os J, Myin-Germeys I and Wichers M. Impact of variation in the BDNF gene on social stress sensitivity and the buffering impact of positive emotions: replication and extension of a gene-environment interaction. ***Eur Neuropsychopharmacol*** 2014; 24(6): 930-8.
