# Supplementary material for: Residential green space and child intelligence and behavior across urban, suburban, and rural areas in Belgium: A longitudinal birth cohort study of twins
Source: PLoS Med. 2020 Aug 18;17(8):e1003213. doi: 10.1371/journal.pmed.1003213 (PMC7446904; doi:10.1371/journal.pmed.1003213)
Supplement: S1 Table — CI, confidence interval; IQR, interquartile range; TIQ, total intelligence quotient; TPIQ, performance intelligence quotient; TVIQ, verbal intelligence quotient. *low versus high. (DOCX) [file pmed.1003213.s004.docx]

| **S1 Table Full model with all covariates of the association between green space in 3000m radius around the residence and intelligence** | | | | | | | | | |
| --- | --- | --- | --- | --- | --- | --- | --- | --- | --- |
|  | **Main model TIQ** | | | **Main model TVIQ** | | | **Main model TPIQ** | | |
|  | **Change in TIQ** | **95%CI** | **P value** | **Change in TVIQ** | **95%CI** | **P value** | **Change in TPIQ** | **95%CI** | **P value** |
| **Urban (n=236)** |  |  |  |  |  |  |  |  |  |
| Boys | 4.1 | 1.0 to 7.2 | 0.01 | 4.6 | 1.7 to 7.6 | 0.003 | 2.3 | -1.3 to 6.0 | 0.21 |
| Age, + 1 year | 1.3 | 0.2 to 2.3 | 0.02 | 0.93 | -0.1 to 1.9 | 0.07 | 1.2 | -0.01 to 2.4 | 0.05 |
| Parental education level* | -9.3 | -12.9 to 5.8 | <.001 | -9.8 | -13.3 to -6.4 | <.001 | -6.5 | -10.5 to -2.4 | 0.002 |
| Neighbourhood household income, +IQR | 0.48 | -1.2 to 2.1 | 0.57 | -0.56 | -2.2 to 1.0 | 0.49 | 1.5 | -0.4 to 3.4 | 0.13 |
| Year of IQ test, + 1 year | -2.2 | -3.6 to -0.9 | 0.002 | -1.5 | -2.8 to -0.2 | 0.02 | -2.2 | -3.7 to -0.6 | 0.008 |
| Green space in 3000m, + IQR | 2.6 | 1.4 to 3.9 | <.001 | 2.2 | 0.9 to 3.4 | 0.0008 | 2.4 | 1.0 to 3.8 | 0.0014 |
|  |  |  |  |  |  |  |  |  |  |
| **Suburban (n=128)** |  |  |  |  |  |  |  |  |  |
| Boys | 0.24 | -5.0 to 5.5 | 0.93 | 2.78 | -2.1 to 7.7 | 0.27 | -1.56 | -7.4 to 4.3 | 0.60 |
| Age, + 1 year | -0.50 | -2.2 to 1.1 | 0.55 | -1.17 | -2.7 to 0.4 | 0.15 | -0.20 | 1.9 to 1.5 | 0.82 |
| Parental education level* | -5.43 | -12 to 1.1 | 0.11 | -6.04 | -12.1 to 0.03 | 0.06 | -5.15 | -12.2 to 1.9 | 0.16 |
| Neighbourhood household income, +IQR | 0.46 | -3.8 to 4.7 | 0.84 | 0.17 | -3.8 to 4.1 | 0.93 | -0.61 | -5.6 to 4.4 | 0.81 |
| Year of IQ test, + 1 year | -0.62 | -2.3 to 1.0 | 0.46 | -1.15 | -2.7 to 0.4 | 0.16 | -0.26 | -2.0 to 1.5 | 0.77 |
| Green space in 3000m, + IQR | 1.51 | -1.2 to 4.2 | 0.27 | 1.50 | -1.1 to 4.1 | 0.27 | 1.40 | -0.9 to 3.7 | 0.24 |
|  |  |  |  |  |  |  |  |  |  |
| **Rural (n=256)** |  |  |  |  |  |  |  |  |  |
| Boys | 0.11 | -3.3 to 3.5 | 0.95 | -0.32 | 3.6 to 2.9 | 0.85 | 1.10 | -2.7 to 4.9 | 0.57 |
| Age, + 1 year | 0.32 | -0.9 to 1.5 | 0.60 | 0.58 | -0.5 to 1.6 | 0.30 | 0.02 | -1.2 to 1.3 | 0.98 |
| Parental education level* | -9.29 | -13.7 to -4.8 | <0.001 | -8.71 | -12.7 to -4.7 | <0.001 | -7.57 | -12.3 to -2.9 | 0.002 |
| Neighbourhood household income, +IQR | -2.12 | -4.1 to -0.1 | 0.04 | -1.73 | -3.6 to 0.1 | 0.07 | -2.18 | -4.3 to -0.1 | 0.04 |
| Year of IQ test, + 1 year | -2.08 | -3.6 to -0.6 | 0.008 | -1.93 | -3.3 to -0.6 | 0.006 | -1.40 | -3.0 to 0.2 | 0.08 |
| Green space in 3000m, + IQR | -1.32 | -3.8 to 1.1 | 0.29 | -0.82 | -3.0 to 1.4 | 0.46 | -1.38 | -4.0 to 1.2 | 0.30 |
|  |  |  |  |  |  |  |  |  |  |

TIQ Total Intelligence Quotient, TVIQ Verbal Intelligence Quotient, TPIQ Performance Intelligence Quotient, CI confidence intervals, IQR interquartile range, *low vs high
