# Supplementary figures and images for: Residential green space and child intelligence and behavior across urban, suburban, and rural areas in Belgium: A longitudinal birth cohort study of twins
Source: PLoS Med. 2020 Aug 18;17(8):e1003213. doi: 10.1371/journal.pmed.1003213 (PMC7446904; doi:10.1371/journal.pmed.1003213)

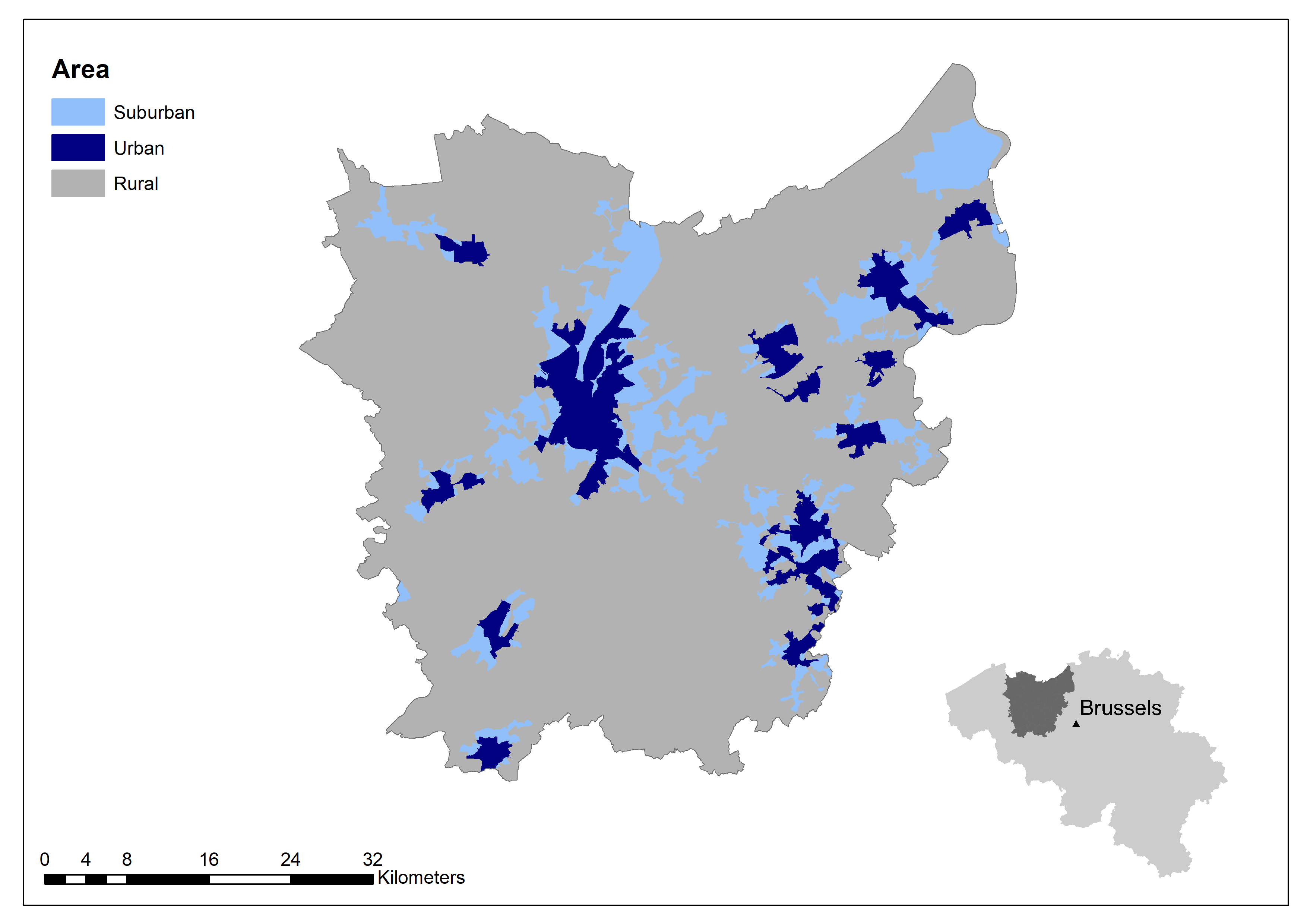

Supplement: S1 Fig — Source: Flemish Government–Department Environment. (TIF) [file pmed.1003213.s005.tif]

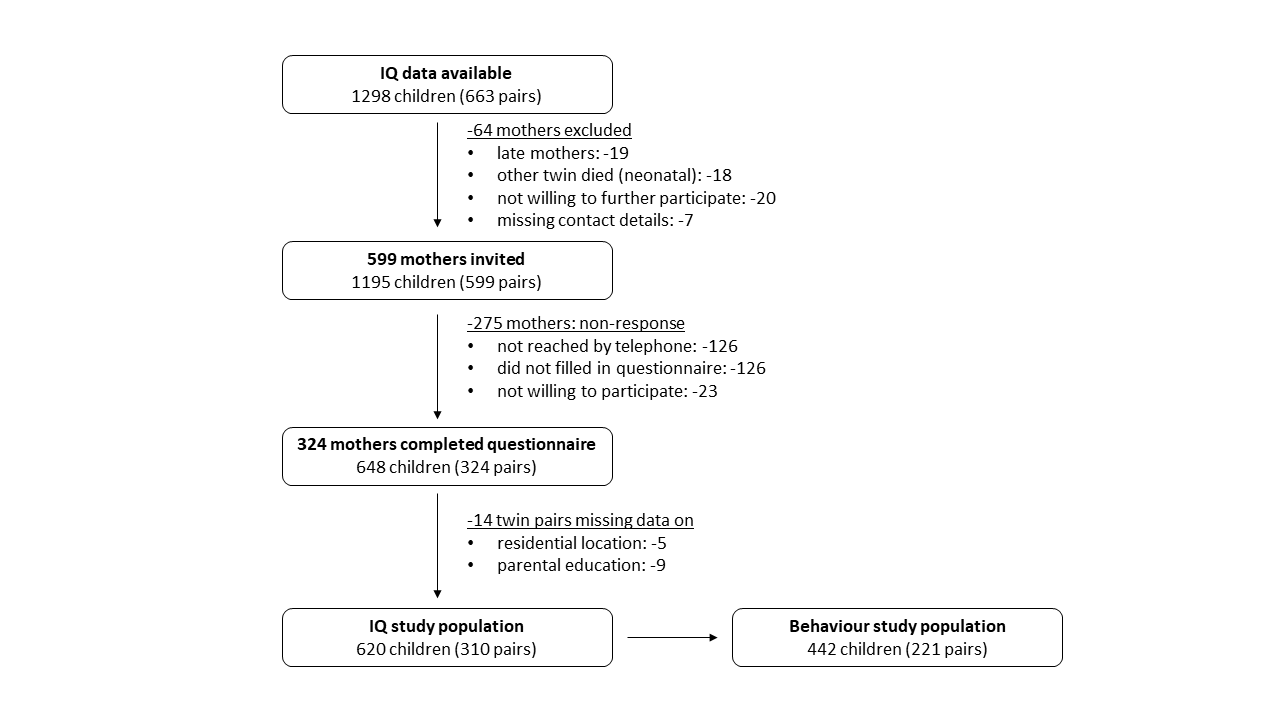

Supplement: S2 Fig — From the children with IQ data available, 599 mothers were invited to complete a questionnaire on residential history. A total of 324 mothers completed the questionnaire. From the twin pairs, we excluded 14 pairs because of missing data on residential location or parental education resulting in a final IQ study sample of 620 children. For a subset of 442 children, aged 6–15 years, data are available for studying behavioral characteristics. IQ, intelligence quotient. (TIF) [file pmed.1003213.s006.tif]
