# Supplementary material for: Residential green space and child intelligence and behavior across urban, suburban, and rural areas in Belgium: A longitudinal birth cohort study of twins
Source: PLoS Med. 2020 Aug 18;17(8):e1003213. doi: 10.1371/journal.pmed.1003213 (PMC7446904; doi:10.1371/journal.pmed.1003213)
Supplement: Attachment — Submitted filename: bijnens.pdf [file pmed.1003213.s012.pdf]

This paper reports on the association between child outcomes and environmental greenness in a sample of twins in East Flanders.

I was asked for a statistical report and I interpret that to include all aspects of the design and conduct of the study.

1 Points of detail

Page 4 Strictly speaking the number excluded for various reasons including missingness is part of the results. I wonder whether a flowchart of this might be better and then reasons could be included for why not all were invited and why not all responded.

Page 7 Was the decision to examine the interaction planned? It does seem a plausible thing to look at, I am not accusing the authors of of data fishing.

Page 7 What does 3000–4000 mean? Is this the annulus defined by those two circles or something else?

Page 8 Perhaps remind the reader that low scores on the behaviour checklist are good?

2 Points of more substance

2.1 The modelling

Buffer zones

The authors have identified green areas within buffer zones of five different radii (page 5). In the results section they present results for different radii for different analyses. In the text from page 7 onwards we have 3000–4000, 3000, 2000–3000, and 3000. However the figures (except for figure 1) present results for each radius. I think we need some explanation for this otherwise the suspicious reader might think the authors have just picked the one for the text which was most striking.

Presentation of the full model

The authors have fitted their model based on theoretical principles which is clearly the correct way to go. Some of the benefit of their study for future researchers will lost because the full results from the models with all the covariates are not presented. This extra information could go into supplementary material if the authors feel it would overload the article.

Missingness

Since I assume that the mothers agreed for their twins to be enrolled in the study I am surprised that when asked to contribute to this particular study only 324 out of 615 did so (53%). Why was this? Are there any characteristics of the mothers that predict non–response? Similarly only 436 children had behaviour scores so we need to know why and what variables predict missingness here too. Some discussion of possible bias would also be helpful.

2.2 The context

What is green?

It is not totally clear to me how arable land is classified or how pasture is classified. Many of the advantages of having green space near one's home do not apply to arable and to a lesser extent to pasture. I assume from hints in the article that only publicly accessible green spaces are relevant but it could easily be clarified for us.

How typical is East Flanders?

I wonder whether we need a bit more detail about what urban and rural areas look like in East Flanders. I do not think anyone is going to try to extrapolate from Gent to Beijing, that would be silly, but suppose public health authorities in Gijón (which I think is a similar size to Gent) wanted to know whether it applied to them? I am not sure what details might help them as this is not my area but perhaps: what do green spaces in Flanders look like, what are they used for by the public, what do the rural areas look like?

Are twins representative?

I know that twins have been the focus of many studies but do we need to see any comment about how typical twins are of singletons?

3 Summary

There is quite a bit of missing information which would enhance the paper. I imagine the authors can readily supply it. There is, as far as I know, no length limit on papers here.

Michael Dewey
