# Supplementary material for: Residential green space and child intelligence and behavior across urban, suburban, and rural areas in Belgium: A longitudinal birth cohort study of twins
Source: PLoS Med. 2020 Aug 18;17(8):e1003213. doi: 10.1371/journal.pmed.1003213 (PMC7446904; doi:10.1371/journal.pmed.1003213)
Supplement: Attachment — Submitted filename: ReviewPLOS Medecine.docx [file pmed.1003213.s013.docx]

Thank you for inviting me to review the manuscript “The importance of residential greenness on intellectual and behavioral development in children living in urban versus rural environments” for PLOS Medicine. The main objective is to analyze the association between environmental green space and intelligence and behavior in a study that includes urban and rural environments across east Flanders in Belgium. I think findings do contribute to existing knowledge nowadays on this topic. However, I have some commentaries about this paper.

This paper has two major contributions; firstly, previous studies investigate the association between greenness and cognition and behavior and this one analyze intelligence assessed by Whechler Intelligence Scale for Children (WISC-R). The existing literature shows its beneficial effects on specific cognitive and behavioral functions, instead of total IQ. That is why this is one of the strengths of the work. However, the affirmation in line 92 of “the effect on intelligence is unknown “sounds if they were completely separate and independent constructs. On the other hand, being the second strength of the study the fact that they analyze differences in two different areas, urban and rural, and taking into account that there are not many evidences in analyzing the associations between greenness and rural areas, more information in this sense it should be added in the introduction.

I have some other minor remarks:

1) Page 3. In the first paragraph, evidences of the impact of air pollution and neurodevelopment are introduced. However, the relationship between the main construct, greenness, and air pollution is not presented. Reduction of air pollution is one of the beneficial consequences of the exposition of green spaces but is not the only one, and there are more biopsychosocial mechanisms that try to explain the beneficial effects. Exposition to green spaces has environmental benefits because it reduces air pollution, air pollution, or even heat. But this idea is explained later on, and it results confusing.

2) Page 3 lines 61-62: “environmental factors may impact these performances and neuroinflammation” neuroinflammation is not explained and developed.

3) Page 5: In relation to WISC-R: I would add more information about such as number of items, max and min…On the other hand, who did it administer? Was it administered by a unique trained person or more than one? in the second case information about inter-evaluator reliability. In line 150 “the total scores of the subscales and..” it is not clear that the subscale are verbal and performance.

4) Page 6 line 152: Achenbach Child behavior Checklist (CBCL) is not in the reference list. Is there a Dutch validated version?

5) Figure 1. A) B) and C) are not in the title of each one of the figure (total intelligence quotient, verbal and performance intelligence quotient. In this figure the reason why two correlations are made is not explained (urban area n=230 and 234; suburban na= 126 and 128 and rural only with n=252).

6) Page 7 line 215: Prenatal greenness exposure data collection is not described in Methods and it is not mentioned neither in Introduction. That is why the analysis between greenness during pregnancy with intelligence is not expected. This information should be added in the respective sections in order to understand this result and the paragraph of the Discussion, page 9 line 254.

7) Page 8 line 228: The first sentence refers to a strength/contribution of the work. I suggest to put it on later on in the Discussion, after explaining the main conclusions. Furthermore, the same idea is repeated in the second paragraph “by our knowledge this is the first study investigating the association between residential greenness and intelligence in children”.

8) In Discussion the results related to behavioral components are not commented. An association is observed in total and externalizing problems (including attention and aggressive behavior). There are evidences in this sense that should be discussed.

9) Before publication a revision of English should be done by a native speaker.
