# Supplementary material for: Residential green space and child intelligence and behavior across urban, suburban, and rural areas in Belgium: A longitudinal birth cohort study of twins
Source: PLoS Med. 2020 Aug 18;17(8):e1003213. doi: 10.1371/journal.pmed.1003213 (PMC7446904; doi:10.1371/journal.pmed.1003213)
Supplement: Attachment — Submitted filename: 200501_response_letter_PlosMed2.docx [file pmed.1003213.s014.docx]

Dear editor,

Thank you very much for giving us the opportunity to revise our manuscript: ***“*The importance of residential green space on intellectual and behavioural development in children living in urban versus rural environments in Belgium; a longitudinal birth cohort study*”***. We would like to thank the reviewers for their thoughtful comments which allow us to improve our manuscript. We carefully addressed all comments. A point by point reply to the comments is given in the document containing the response to reviewers. We would like to give our appreciation for these constructive and high quality reviews.

We hope that with these modifications, our manuscript will be acceptable for publication in *Plos Medicine*.

We are looking forward to hearing from you.

Sincerely yours,

Tim Nawrot and Esmée Bijnens on behalf of all authors

**Response to comments:**

Requests from the editors:

**1. Please revise your title according to PLOS Medicine's style. Your title must be nondeclarative and not a question. It should begin with main concept if possible. "Effect of" should be used only if causality can be inferred, i.e., for an RCT. Please place the study design ("A randomized controlled trial," "A retrospective study," "A modelling study," etc.) in the subtitle (ie, after a colon). In addition, as the clarity around the use of ‘greenness’ has been raised by referees, please also consider this and please add the country setting**.

We revised our title (page 1 lines 5-8):” *The importance of residential green space on intellectual and behavioural development in children living in urban versus rural environments in Belgium; a longitudinal birth cohort study.”*

**2. Abstract – Please start the ‘Methods and Findings’ section with more general background about the study rather than the intelligence scale used (focus should be more on how selection took place, numbers, dates, locations and some brief demographic information); please add a sentence on the limitations of the study as the final sentence or two of the Methods and Findings section.**

We added extra information to the ‘Methods and Findings’ section of the abstract (page 2, line 37 and 49):

*“This study includes 614 children and is part of the East Flanders Prospective Twin Survey (EFPTS), a registry of multiple births in the province of East Flanders, Belgium.”*

*“A limitation of this study is that no information was available on school location.”*

**3. Data - PLOS Medicine requires that the de-identified data underlying the specific results in a published article be made available, without restrictions on access, in a public repository or as Supporting Information at the time of article publication, provided it is legal and ethical to do so. Note Corresponding authors cannot be points of contact for data requests. Please see the policy at** [**http://journals.plos.org/plosmedicine/s/data-availability**](http://journals.plos.org/plosmedicine/s/data-availability) **and FAQs at** [**http://journals.plos.org/plosmedicine/s/data-availability#loc-faqs-for-data-policy**](http://journals.plos.org/plosmedicine/s/data-availability#loc-faqs-for-data-policy)

Data used in this article belongs to the East Flanders Prospective Twin Survey and cannot be publicly shared. We provided all information necessary for interested researchers to apply to gain access to the data in the data availability statement (page 15 line 469):

*“Green space data (CORINE land cover) used in this study can be obtained from the European Environment Agency (EEA) (*[*https://land.copernicus.eu/pan-european/corine-land-cover*](https://land.copernicus.eu/pan-european/corine-land-cover)*). Air pollution data used in this study can be obtained from IRCEL (*[*https://www.irceline.be/en*](https://www.irceline.be/en)*). Access to, and use of summarized health data of study participants requires submission of a research proposal to the East Flanders Prospective Twin Survey, Ghent University, contact email,* [*twins@uzgent.be*](mailto:twins@uzgent.be)*.”*

**4. Line 109 – please ensure all questionnaires are provided (and translated to English, as needed) as Supp Files and provide a callout in the methods.**

*We added a callout in the methods (page 5, line 135): “Of the 663 pairs, we invited 599 eligible mothers to complete a questionnaire (S1 Text) on residential history.*

**5. Line 113 – what is the ‘Child Behaviour Checklist’**

Information on the Child Behaviour Checklist is available in section *“Assessment of intelligence and behaviour during childhood”* of the methods.

We agree that line 113 might be confusing to readers to mention the Child Behaviour Checklist early in the methods without extra information and we therefore removed the sentence “*Their parents filled out the Child Behaviour Checklist (CBCL).*” We added on lines 211-216 on page 7 detailed information: *“Parents were asked to answer each item in 3 levels: 0=not true, 1=sometimes/somewhat true, 2=very true/often true, with higher score indicating more adverse behaviour. The sum of all questions served as a global score for children's problematic behaviour (the total problems behaviour score). Separate scores were calculated for internalizing problems and externalizing problems. CBCL T-scores equal to 64 or higher on the internalizing, externalizing, or total problem scales are considered in the clinical range* *(Achenbach et al., 2001).”*

**6. Line 228 – remove ‘till now’ and instead replace with ‘to our knowledge this is the first…’
Be cautious around overstating findings (eg in conclusion…’ is especially’ ….may be would be better as this isn’t a trial)**

*L228 Till now no studies on intelligence and residential greenness were reported.*

*We removed this first sentence of the discussion on request of reviewer 3 and we kept the sentence in the second paragraph (line 325 on page 11):* “By our knowledge this is the first study investigating the association between residential green space and intelligence in children”

We added later in the discussion (line 442 on page 14): “An *important contribution of this paper is that to our knowledge this is the first study on intelligence and residential green space.”*

*We replaced as requested* (line 442 on page 14 *“*Our results indicate that residential green space may be beneficial for intellectual and behavioural development of children living in an urban environment.”

**7. Did your study have a prospective protocol or analysis plan? Please state this (either way) early in the Methods** **section.**

**a) If a prospective analysis plan (from your funding proposal, IRB or other ethics committee submission, study protocol, or other planning document written before analyzing the data) was used in designing the study, please include the relevant prospectively written document with your revised manuscript as a Supporting**

**Information file to be published alongside your study, and cite it in the Methods section. A legend for this file should be included at the end of your manuscript.**

This study is part of a project entitled “Residential surrounding greenness, traffic exposure and maternal stress as determinants of neurocognitive and neurobehavioural development during the first decade of life in twins and triplets”. Funding was received from the Marguerite-Marie Delacroix foundation in 2016. The current study can be found in the funding proposal (point number one of the specific aims). The statistical plan is now included as a supplement.

We mention in the methods (page 5, line 151): “*The method and study design used in this study were described before the analyses were conducted, and the prospective analysis plan is present in supplement (S2 Text).”*

We added the following legend:” A *priori statistical analysis plan”*

**b) If no such document exists, please make sure that the Methods section transparently describes when analyses were planned, and when/why any data-driven changes to analyses took place.

c) In either case, changes in the analysis-- including those made in response to peer review comments-- should be identified as such in the Methods section of the paper, with rationale.**

**8. Please ensure that the study is reported according to the STROBE guideline, and include the completed STROBE checklist as Supporting Information. Please add the following statement, or similar, to the Methods: "This study is reported as per the Strengthening the Reporting of Observational Studies in Epidemiology (STROBE) guideline (S1 Checklist)."**

**The STROBE guideline can be found here: http://www.equator-network.org/reporting-guidelines/strobe/

When completing the checklist, please use section and paragraph numbers, rather than page numbers.**

The study is reported according to the STROBE guideline and the STROBE checklist is included as Supporting Information.

We added to the methods (page 5, line 150): "*This study is reported as per the Strengthening the Reporting of Observational Studies in Epidemiology (STROBE) guideline (S1 Checklist)."*

**Response to comments reviewers:**

Reviewer #1, Michael Dewey:

This paper reports on the association between child outcomes and environmental greenness in a sample of twins in East Flanders. I was asked for a statistical report and I interpret that to include all aspects of the design and conduct of the study.

**1 Points of detail**

**Page 4 Strictly speaking the number excluded for various reasons including missingness is part of the results. I wonder whether a flowchart of this might be better and then reasons could be included for why not all were invited and why not all responded.**

We added to the methods (page 5, line 134):

“*In 1992, and between 1996 and 1999, we assessed the intelligence in 663 twin pairs at a mean age of 10.4 years (age range 7-15) using the Wechsler Intelligence Scale for Children-Revised (WISC-R). Of the 663 pairs, we invited 599 eligible mothers to complete a questionnaire (S1 Text) on residential history. In total 64 mother/twin pairs were lost for the present retrospective study: mothers with missing contact details (n=7), mothers who indicated not willing to further participate (n=20), late mothers (n=19), and twin pairs of which one of the twins died (still born or neonatal, n=18). The questionnaire was completed by 324 mothers (54%) and 275 mothers must be classified as non-respondents, i.e. 126 could not be reached, 126 did not fill out the questionnaire, and 23 indicated not willing to participate. We excluded 14 twin pairs from our analysis because of missing data [on residential location (n=5) and parental educational level (n=9)]. The number of children included in our analysis was 620 children (310 twin pairs). From a subset of 442 children (221 twin pairs), aged 6-15 years, data is available on behavioural analysis. An overview is presented in the flowchart as supplement (S2 Fig).”*

**S2 Fig flowchart.** From the children with IQ data available, 599 mothers were invited to complete a questionnaire on residential history. 324 mothers completed the questionnaire. From the twin pairs, we excluded 14 pairs because of missing data on residential location or parental education resulting in a final IQ study sample of 620 children. For a subset of 442 children, aged 6-15 years, data is available for studying behavioural characteristics.

**Page 7 Was the decision to examine the interaction planned? It does seem a plausible thing to look at, I am not accusing the authors of data fishing**.

This study is part of the project entitled “Residential surrounding greenness, traffic exposure and maternal stress as determinants of neurocognitive and neurobehavioural development during the first decade of life in twins and triplets”. Funding was received from the Marguerite-Marie Delacroix foundation in 2016. The current study can be found in the funding proposal (point number one of the specific aims).

We mention in the methods (page 5, line 151): “*The method and study design used in this study were described before the analyses were conducted, and the prospective analysis plan is present in supplement (S2 Text).”*

**Page 7 What does 3000-4000 mean? Is this the annulus defined by those two circles or something else?**

We clarified the sentence (page 9, line 281): “*In urban areas, residential green space within a 1000 to 5000m buffer is significantly associated with total - and performance IQ, while the association between urban greenness and verbal IQ was significant in a 1000 to 4000m buffer and not in a 500m and 5000m buffer.”*

**Page 8 Perhaps remind the reader that low scores on the behaviour checklist are good?**

We added to the methods (lines 211-216 on page 7): *“Parents were asked to answer each item in 3 levels: 0=not true, 1=sometimes/somewhat true, 2=very true/often true, with higher score indicating more adverse behaviour. The sum of all questions served as a global score for children's problematic behaviour (the total problems behaviour score). Separate scores were calculated for internalizing problems and externalizing problems. CBCL T-scores equal to 64 or higher on the internalizing, externalizing, or total problem scales are considered in the clinical range* *(Achenbach et al., 2001).”*

**2 Points of more substance**

**2.1 The modelling**

**Buffer zones**

**The authors have identified green areas within buffer zones of five different radii (page 5). In the results section they present results for different radii for different analyses. In the text from page 7 onwards we have 3000-4000, 3000, 2000-3000, and 3000. However, the figures (except for figure 1) present results for each radius. I think we need some explanation for this otherwise the suspicious reader might think the authors have just picked the one for the text which was most striking.**

We want to be transparent on all data and therefore we show all the results for the different radii in the figures. We now stress in the text also the non-significant results.

We rewrote the results as follows (page 9, line 281; page 10, line 310): *“In urban areas, residential green space within a 1000 to 5000m buffer is significantly associated with total - and performance IQ, while the association between urban greenness and verbal IQ was significant in a 1000 to 4000m buffer and not in a 500m and 5000m buffer. Taking the percentage green within a 3000m as a reference we found that for an IQR (3.3%) increase in green space within a 3000m radius from the residential address is associated with a 2.6 point higher (95% CI: 1.4 to 3.9; p<0.0001) total IQ, a 2.2 point higher (95% CI: 0.9 to 3.4; p= 0.008) verbal IQ, and a 2.4 point higher (95% CI: 1.0 to 3.8; p=0.0014) performance IQ among children residing in an urban environment*.”

*“Similarly, higher residential green space is associated with a reduction in behavioural problems in children living in an urban area, while no effect was noted in children living in a rural or suburban area (Figure 4). Percentage of residential green space within a 2000m and a 3000m radii from the residential address in an urban environment was significantly associated with a reduction in total problem behaviour, green space within a 1000m to 5000m radius is significantly associated with a reduction in externalizing problem behaviour (including attention problems and aggressive behaviour) and greenspace in a 3000m radius is associated with lower internalizing problem behaviour (including anxiety and withdrawal).”*

We added to the discussion (page 14, line 418)*:” The association between green space and the outcomes IQ and behaviour was investigated in several radius distances around the residential address. We found that the positive impact of green in an urban setting for both IQ and behavioural scores depended on the percentage green within 2000 and 3000m. It was unfeasible to choose buffers smaller than 500m given the resolution of CORINE land cover data.”*

**Presentation of the full model**

**The authors have fitted their model based on theoretical principles which is clearly the correct way to go. Some of the benefit of their study for future researchers will lost because the full results from the models with all the covariates are not presented. This extra information could go into supplementary material if the authors feel it would overload the article.**

We added to the results (page 10, line 288):” The full results of the model with all the covariates are shown in supplement (S1 Table)”.

| **S1 Table Full model with all covariates of the association between green space in 3000m radius around the residence and intelligence** | | | | | | | | | |
| --- | --- | --- | --- | --- | --- | --- | --- | --- | --- |
|  | **Main model TIQ** | | | **Main model TVIQ** | | | **Main model TPIQ** | | |
|  | **Change in TIQ** | **95%CI** | **P value** | **Change in TVIQ** | **95%CI** | **P value** | **Change in TPIQ** | **95%CI** | **P value** |
| **Urban (n=236)** |  |  |  |  |  |  |  |  |  |
| Boys | 4.1 | 1.0 to 7.2 | 0.01 | 4.6 | 1.7 to 7.6 | 0.003 | 2.3 | -1.3 to 6.0 | 0.21 |
| Age, + 1 year | 1.3 | 0.2 to 2.3 | 0.02 | 0.93 | -0.1 to 1.9 | 0.07 | 1.2 | -0.01 to 2.4 | 0.05 |
| Parental education level* | -9.3 | -12.9 to 5.8 | <.0001 | -9.8 | -13.3 to -6.4 | <.0001 | -6.5 | -10.5 to -2.4 | 0.002 |
| Neighbourhood household income, +IQR | 0.48 | -1.2 to 2.1 | 0.57 | -0.56 | -2.2 to 1.0 | 0.49 | 1.5 | -0.4 to 3.4 | 0.13 |
| Year of IQ test, + 1 year | -2.2 | -3.6 to -0.9 | 0.002 | -1.5 | -2.8 to -0.2 | 0.02 | -2.2 | -3.7 to -0.6 | 0.008 |
| Green space in 3000m, + IQR | 2.6 | 1.4 to 3.9 | <.0001 | 2.2 | 0.9 to 3.4 | 0.0008 | 2.4 | 0.9 to 3.8 | 0.001 |
|  |  |  |  |  |  |  |  |  |  |
| **Suburban (n=128)** |  |  |  |  |  |  |  |  |  |
| Boys | 0.24 | -5.0 to 5.5 | 0.93 | 2.78 | -2.1 to 7.7 | 0.27 | -1.56 | -7.4 to 4.3 | 0.60 |
| Age, + 1 year | -0.50 | -2.2 to 1.1 | 0.55 | -1.17 | -2.7 to 0.4 | 0.15 | -0.20 | 1.9 to 1.5 | 0.82 |
| Parental education level* | -5.43 | -12 to 1.1 | 0.11 | -6.04 | -12.1 to 0.03 | 0.06 | -5.15 | -12.2 to 1.9 | 0.16 |
| Neighbourhood household income, +IQR | 0.46 | -3.8 to 4.7 | 0.84 | 0.17 | -3.8 to 4.1 | 0.93 | -0.61 | -5.6 to 4.4 | 0.81 |
| Year of IQ test, + 1 year | -0.62 | -2.3 to 1.0 | 0.46 | -1.15 | -2.7 to 0.4 | 0.16 | -0.26 | -2.0 to 1.5 | 0.77 |
| Green space in 3000m, + IQR | 1.51 | -1.2 to 4.2 | 0.27 | 1.50 | -1.1 to 4.1 | 0.27 | 1.40 | -0.9 to 3.7 | 0.24 |
|  |  |  |  |  |  |  |  |  |  |
| **Rural (n=256)** |  |  |  |  |  |  |  |  |  |
| Boys | 0.11 | -3.3 to 3.5 | 0.95 | -0.32 | 3.6 to 2.9 | 0.85 | 1.10 | -2.7 to 4.9 | 0.57 |
| Age, + 1 year | 0.32 | -0.9 to 1.5 | 0.60 | 0.58 | -0.5 to 1.6 | 0.30 | 0.02 | -1.2 to 1.3 | 0.98 |
| Parental education level* | -9.29 | -13.7 to -4.8 | <0.0001 | -8.71 | -12.7 to -4.7 | <0.0001 | -7.57 | -12.3 to -2.9 | 0.002 |
| Neighbourhood household income, +IQR | -2.12 | -4.1 to -0.1 | 0.04 | -1.73 | -3.6 to 0.1 | 0.07 | -2.18 | -4.3 to -0.1 | 0.04 |
| Year of IQ test, + 1 year | -2.08 | -3.6 to -0.6 | 0.008 | -1.93 | -3.3 to -0.6 | 0.006 | -1.40 | -3.0 to 0.2 | 0.08 |
| Green space in 3000m, + IQR | -1.32 | -3.8 to 1.1 | 0.29 | -0.82 | -3.0 to 1.4 | 0.46 | -1.38 | -4.0 to 1.2 | 0.30 |
|  |  |  |  |  |  |  |  |  |  |

TIQ Total Intelligence Quotient, TVIQ Verbal Intelligence Quotient, TPIQ Performance Intelligence Quotient, CI confidence intervals, IQR interquartile range, *low vs high

**Missingness**

**Since I assume that the mothers agreed for their twins to be enrolled in the study I am surprised that when asked to contribute to this particular study only 324 out of 615 did so (53%). Why was this? Are there any characteristics of the mothers that predict non-response? Similarly only 436 children had behaviour scores so we need to know why and what variables predict missingness here too. Some discussion of possible bias would also be helpful.**

To clarify this point we added a flow chart as supplementary file (S2 Fig).

**S2 Fig flowchart.** From the children with IQ data available, 599 mothers were invited to complete a questionnaire on residential history. 324 mothers completed the questionnaire. From the twin pairs, we excluded 14 pairs because of missing data on residential location or parental education resulting in a final IQ study sample of 620 children. For a subset of 442 children, aged 6-15 years, data is available for studying behavioural characteristics.

The IQ assessment was performed in 1992 and between 1996 and 1999 (Jacobs *et al*., 2000, Antoniou *et al*., 2013). The mothers were only recently invited (February 2017 and March 2018) to complete a retrospective questionnaire on residential history.

We added to the methods (page 5, line 139): “*The questionnaire was completed by 324 mothers (54%) and 275 mothers must be classified as non-respondents, i.e. 126 could not be reached, 126 did not fill out the questionnaire, and 23 indicated not willing to participate. We excluded 14 twin pairs from our analysis because of missing data [on residential location (n=5) and parental educational level (n=9)]. The number of children included in our analysis was 620 children (310 twin pairs). From a subset of 442 children (221 twin pairs), aged 6-15 years, data is available on behavioural analysis. An overview is presented in the flowchart as supplement (S2 Fig).”*

We compared the characteristics of those that we were able to collect information on residential history compared with non-responders. Children included in the study differed slightly from those of which we were not able to collect data in terms of parental educational level, IQ, and externalizing behaviour (see table below). However, given that the differences between responders and non-responders were rather small, it is unlikely that this would have had a major impact on the results.

We added this paragraph on page 5 and line 145 of the methods just after the description of the participant selection.

The reason that we had different numbers on intelligence and behaviour assessment is because the twins were originally recruited in two sampling frames (behaviour and intelligence). This is described in Jacobs *et al.* (2002). This reference is added to the methods. In the first, 1436 twins aged 6–17 years were selected from the EFPTS. Their parents were asked to fill in the Child Behaviour Checklist (CBCL). Seven hundred and sixty parents (53% of the total available parents) participated. In the second sampling frame, twins from the EFPTS born between September 1982 and December 1991 were invited to participate. In total, 663 twin pairs aged 8–14 years were tested. Of the 663 pairs, 376 had also participated in the first study in which the CBCL had been used.

*Jacobs N, Rijsdijk F, Derom C, Danckaerts M, Thiery E, Derom R, et al. Child psychopathology and lower cognitive ability: a general population twin study of the causes of association. Mol Psychiatry. 2002;7(4):368–74.*

| **Table 1. Study population characteristics** | | |  | |  |
| --- | --- | --- | --- | --- | --- |
| **Characteristic** | **Responders n = 620** | **Non-responders n = 547** | | **p-Value** |  |
|  |  |  | |  |  |
| Sex, male | 312 (50.3) | 276 (50.5) | | 0.96 |  |
| Birth year | 1986 ± 2.8 | 1987 ± 2.7 | | 0.006 |  |
| Zygosity - Chorionicity |  |  | | 0.17 |  |
| Dizygotic-Dichorionic | 356 (57.4) | 320 (58.5) | |  |  |
| Monozygotic-Dichorionic | 106 (17.1) | 65 (11.9) | |  |  |
| Monozygotic-Monochorionic | 158 (25.5) | 162 (29.6) | |  |  |
| Parental education |  |  | | 0.0009 |  |
| Low | 18 (5.8) | 32 (13) | |  |  |
| Middle | 102 (32.9) | 97 (39.0) | |  |  |
| High | 190 (61.3) | 118 (47.8) | |  |  |
| Missing | 0 | 28 | |  |  |
|  |  |  | |  |  |
|  |  |  | |  |  |
| **Intelligence (WISC-R)** | **n = 620** | **n = 547** | |  |  |
| Age at investigation | 11.0 ± 1.8 | 10.7± 1.8 | | 0.03 |  |
| Year of investigation | 1998 ± 1.6 | 1998 ± 1.5 | | 0.05 |  |
| Intelligence score (IQ) |  |  | |  |  |
| Total IQ | 105.3± 14.2 | 99.7± 14.3 | | <0.0001 |  |
| Verbal | 106.2 ± 13.5 | 100.6 ± 14.7 | | <0.0001 |  |
| Performance | 103.2 ± 15.2 | 99.8 ± 14.8 | | <0.0001 |  |
|  |  |  | |  |  |
| **Behaviour (CBCL)** | **n= 442** | **n= 222** | |  |  |
| Age at investigation | 8.5 ± 2.2 | 8.2 ± 2.1 | | 0.34 |  |
| Year of investigation | 1994 ± 0.5 | 1994 ± 0.5 | | 0.39 |  |
| CBCL score |  |  | |  |  |
| Total | 46.5 ± 9.9 | 47.6 ± 9.8 | | 0.13 |  |
| Externalizing | 46.3 ± 9.9 | 48.0 ± 9.8 | | 0.02 |  |
| Internalizing | 47.7 ± 9.4 | 48.4 ± 9.2 | | 0.29 |  |
| Data presented are means ± standard deviation or number (percentage). | | |  | |  |

**2.2 The context**

**What is green?**

**It is not totally clear to me how arable land is classified or how pasture is classified. Many of the advantages of having green space near one's home do not apply to arable and to a lesser extent to pasture. I assume from hints in the article that only publicly accessible green spaces are relevant but it could easily be clarified for us.**

Green space does not include agricultural areas (arable land, permanent crops, pasture and heterogenous agricultural areas).

Indeed, literature on green space around schools and academic performance shows mixed results (Browning et al., 2019): Although positive associations were observed for three types of green space: green land cover (45% of the findings), greenness (32%) and tree cover (30%) whereas no positive findings were found for agriculture, grass, or shrub cover. We added this to the introduction page 4 and line 94.

We now clarified in the method section (page 6, line 181): *“Semi-natural-, forested -, blue- and urban green areas (green space) in several radius distances (5000, 4000, 3000, 2000, 1000, and 500m) around the residential address during childhood and during pregnancy were estimated based on CORINE Land Cover 1990 (European Environment Agency). Green space does not include agricultural areas.”*

**How typical is East Flanders?**

**I wonder whether we need a bit more detail about what urban and rural areas look like in East Flanders. I do not think anyone is going to try to extrapolate from Gent to Beijing, that would be silly, but suppose public health authorities in Gijón (which I think is a similar size to Gent) wanted to know whether it applied to them? I am not sure what details might help them as this is not my area but perhaps: what do green spaces in Flanders look like, what are they used for by the public, what do the rural areas look like?**

We added to the methods (page 5, line 129): *“Similar to the rest of Flanders, this province is densely populated with strong urban metropolitan areas and includes a lot of arable land. The capital Ghent has many parks spread throughout the city, readily accessible or within close proximity to home for most residents (Van Hecke et al., 2017). Parks in Flanders are used mostly as a place to relax rather than to engage in intensive sports (Van Hecke et al., 2017; Van Dyck et al., 2013). “*

**Are twins representative?**

**I know that twins have been the focus of many studies but do we need to see any comment about how typical twins are of singletons?**

We added to the discussion (page 13, line 402): *“Our results were observed in twins. We do not expect that twins have a different exposure or react differently to green space than singletons. Studies show that twins score 4 point lower on an IQ test than singletons (Voracek et al., 2008). This difference is partially attributed to difference in intrauterine growth (Voracek et al., 2008), nutrient deficiency, and shorter gestational length of twins (Eriksen et al., 2012). In another study however no evidence on cognitive differences was found between adult twins and their non-twin siblings (Posthuma et al., 2000). Besides lower birth weight is a causal risk factor for child problem behaviour (Van Os et al., 2001). However, a study comparing the developmental trajectories of behavioural problems in 6- to 12-year-old* twins *and* singletons*, confirm the generalizability with regard to externalizing problems in childhood of twin studies to singleton populations (Robbers et al., 2010). Therefore, we expect no large differences in regard to singletons.”*

**3 Summary**

**There is quite a bit of missing information which would enhance the paper. I imagine the authors can readily supply it. There is, as far as I know, no length limit on papers here.**

We thank the reviewer for the constructive comments and in the revised version we clarified and extended on the concerns.

Reviewer #2:

This study is among the first studies on IQ and greenspace, and is among the few existing studies on behavioural problems and greenspace. After revision, it may be considered for publication. My specific comments and recommendations are outlined below

1. **L67-69: The Authors never explain what is greenness, and instead, go to green spaces right away. These are two different concepts, which should be at least briefly mentioned. Greenness refers to general degree of vegetation while by green spaces, structured vegetation in parks, forests, meadows, etc. is meant. Greenness is typically satellite-derived and green spaces are extracted from land use/land cover datasets (if these data are not self-reported, of course).**

We now changed as requested “greenness” into “green space” throughout the manuscript.

We added a description of the different concepts to the introduction (page 3, line 72): “**Greenness refers to the vegetation level, while** green spaces are covered partly by grass, trees, or other vegetation, and include city parks, community gardens, sports ﬁelds, as well as natural and forested areas in rural environments [8] (Taylor and Hochuli, 2017).”

2. **L73-80: Talking about greenspace and academic performance, Authors ignore negative findings, as summarized by** [**https://www.ncbi.nlm.nih.gov/pmc/articles/PMC6388261/**](https://www.ncbi.nlm.nih.gov/pmc/articles/PMC6388261/)**. Moreover, the analysis of Wu et al. had methodological shortcomings, judging by the re-analysis of the data (**[**https://www.sciencedirect.com/science/article/pii/S0169204618303086**](https://www.sciencedirect.com/science/article/pii/S0169204618303086)**).**

Thanks for this comment. In the revised version we made efforts for a balanced introduction and discussion.

We now removed the study of Wu et al. from the introduction and replaced this with (page 4 and line 94):

“*The literature on green space around schools and academic performance shows mixed results (Browning et al., 2019). Although positive associations were observed for three types of green space: green land cover (45% of the findings), greenness (32%) and tree cover (30%) whereas no positive findings were found for agriculture, grass, or shrub cover.”*

We added the replication (Browning *et al.,* 2018) of the study of Wu et al. to the discussion (page 11, line 334):

*“In addition, based on school-level student performance data from 905 public schools in Massachusettes, the second most tree-covered state, collected between 2006 and 2012 (n= 6333), the effect of surrounding greenness on the school performance was investigated [11]. The results showed that children (age 8-9 years) with higher exposure to greenness show better performance in both English and Math. However, this study was replicated in Chicago, in an* area with low tree coverage, *and found no convincing evidence for a positive relationship between residential greenness and academic performance (Browning et al., 2018). The authors suggest that a main reason may be that greenness, the general degree of vegetation, based on NDVI fails to distinguish between vegetation types, such as grasses and trees. In connection to this Kweon et al. found a positive association between tree cover in the school surrounding and academic performance, but grass and shrubs negatively correlate with performance (Kweon et al., 2017).”*

3. **L69-71: Pathways explaining effects of green spaces/greenness are outlined here** [**https://pubmed.ncbi.nlm.nih.gov/28672128-exploring-pathways-linking-greenspace-to-health-theoretical-and-methodological-guidance/**](https://pubmed.ncbi.nlm.nih.gov/28672128-exploring-pathways-linking-greenspace-to-health-theoretical-and-methodological-guidance/) **and here** [**https://pubmed.ncbi.nlm.nih.gov/24387090-nature-and-health/**](https://pubmed.ncbi.nlm.nih.gov/24387090-nature-and-health/)**.**

We now moved the underlying pathways (also in connection with comment number 1 from Reviewer 3) to the introduction (page 3, line 74): “*Green space provide environmental benefits by reducing air and noise pollution. Higher residential green space is associated with lower ambient air pollution [27] and noise exposure [28]. Both have been associated with diminished cognitive development [29-32]. Furthermore, from a health perspective, green space promotes physical activity and stress reduction [9] [33, 34]. Apart from the promotion of physical activity, green space in the living environment could also lead to more social contacts [36]. In addition, higher levels of neighbourhood green space were associated with significantly lower levels of symptoms of depression, anxiety and stress. [40] Besides self-reported stress, also salivary cortisol, an indicator of chronic stress, is negatively associated with exposure to greenness in the living environment [41]. Higher residential green space during pregnancy is even associated with longer telomere length in placental tissue, an important marker of ageing [42]. Finally, according to the biodiversity hypothesis of health, the reduced contact with a natural environment and biodiversity is associated with adverse implications for the human microbiome and its immunomodulating capacity [43]. Microbial input from the environment may drive brain regulation and is a possible major component of the beneficial effect of greenness [44]. Several animal studies suggest that the human microbiome may have profound effects on brain development and linked the gut microbiota to neurodevelopmental processes and behavior [45-47]. Natural space-induced environmental biodiversity has been related to enriched skin and gut microbiota that in turn has been shown to improve brain development, probably through regulation of immune response [50].”*

We condensed this part of the discussion and refer to the study of Markevych et al. (2017) (page 1, line 396): “*The underlying pathways responsible for the positive effect of residential green space exposure on intelligence is not fully understood, but several mechanisms are considered. First, green spaces provide environmental benefits (e.g. reducing exposure to air pollution, noise and heat). Moreover, green spaces encourage health-promoting activities and facilitating social cohesion. A third potential domain linking greenspace to health is the ability of greenspace to restore capacities (e.g. attention restoration and physiological stress recovery). (Markevych et al., 2017)”*

**4. L106: Please correct the reference. It is Supplement Figure 1, not Figure 1.**

We correct the reference to Supplement figure 1*: “The province East Flanders is located in the North-West of Belgium and the capital Ghent is central located. Besides Ghent and a few urban areas most of the province is rural (S1 Fig).”*

**5. L104: Just to make it clear: children born from 1964 were included into the analysis? It does not seem so from the further text.**

To avoid confusion, we adapted this sentence in the methods (page 5, line 125): *“~~The East Flanders Prospective Twin Survey (EFPTS) is a population based register of multiple births in the province of East Flanders (Belgium) since 1964 [19].~~ This study includes 614 children born between 1980 and 1991. This subgroup is part of the East Flanders Prospective Twin Survey (EFPTS), a registry of multiple births in the province of East Flanders, Belgium. Since its start in 1964, over 10,000 twin-pairs have been registered [19] (Derom et al., 2019).”*

**6. L121: A very brief explanation that monochorionic twins share the same placenta would be nice for readers without medical background.**

L121: Zygosity was determined by sequential analysis based on sex, choriontype, blood group determined on umbilical cord blood, placental alkaline phosphatase, and since 1982, DNA fingerprints [21]. After DNA-fingerprinting, a zygosity probability of 99.9% was reached.

We added (page 6, line 163): *“Based on chorionicity, monozygotic twins where further divided into two groups, monozygotic dichorionic and monozygotic monochorionic. Monochorionic twins share only one placenta (Loos et al. 2009)*.”

**7. L133-134: Was complete residential history unknown? At least, time lived at the current address? How about moving during pregnancy? All this may have caused exposure misclassification.**

We have information on time lived at the current address and even about moving during pregnancy and the residential address before pregnancy. 55% of the children lives still at their birth address with no differences according to urbanisation type (respectively 60%, 41% and 57% in urban, suburban and rural). Overall, 86% of the mothers did not move during pregnancy (respectively 89%, 87% and 84% in urban, suburban and rural). The corresponding percentages for living more than 10 years (or the whole life in case the child < 10 years) on the same address was 67% (respectively 68%, 53% and 74% in urban, suburban and rural).

We added the variable (=living minimum 10y at the current address or not) to the model. It was not significant (p=0.38) and this additional adjustment did not alter the reported associations. Even when we investigate the association between green space and IQ in a subset of children (n=144) living minimum 10 years at the current address. The results remained significant (see figure below). We included this in the manuscript as a sensitivity analysis.

We added to the methods (page 8, line 248): “As a sensitivity analysis, we investigated the association between green space and IQ in a subset of children (n=144) living minimum 10 years (or their whole life if the age of the child <10 years old at the current address*.*”

We added to the results (page 10, 298): “*The results remain similar in a subpopulation of children (n=144) living minimum 10 years at the current address (S4 Fig).”*

**S4 Fig Estimated change in intelligence quotient (IQ) for an IQR increase in green space in a 1000-5000m radius around the residence in children living in an urban (n=236) area and in a subgroup of children living minimum 10 years (or their whole life if the age of the child <10 years old) at their current address (n=144).** Adjusted for sex, age, parental education, neighbourhood household income, year of IQ test, and zygosity and chorionicity.

**8. L138-141:**

**8.1 How did this correspond to the years of birth and IQ assessment? Please provide the information on when the children were born and tested. Land cover did not remain absolutely the same over decades.**

We clarified this in the revised methods (line 125, page 5): “*This study includes 620 children born between 1980 and 1991”* and (line 134, page 5) *“In 1992 and between 1996 and 1999, we assessed intelligence in 663 twin pairs at a mean age of 10.4 years (age range 7-15) using the Wechsler Intelligence Scale for Children-Revised (WISC-R)”.* We adjusted for year of investigation and birth year in the models.

The CORINE land cover data were from 1990. No earlier satellite data on land-use were available. However, green spaces calculated based on CORINE land cover is highly correlated even over a decade. See table below.

We mention this in the limitation section of the discussion (page 13, line 415): *“Second, the twins were born from 1980 till 1991, however to determine residential green space during pregnancy we used data from 1990 as no earlier satellite data were available. However strong correlations have been shown over time between periods of one decade [42].”*

| **Table Correlation coefficient between residential green space based on CORINE land cover 1990 and green space based on CORINE land cover 2000 (n=620)** | | | | | |
| --- | --- | --- | --- | --- | --- |
| **Buffer sizes** | **1000** | **2000** | **3000** | **4000** | **5000** |
| Pearson correlation,  p-value | 0.99  <0.0001 | 0.99  <0.0001 | 0.99  <0.0001 | 0.99  <0.0001 | 1.00  <0.0001 |

**8.2Furthermore, why not smaller buffers were considered? At least 500-m buffer would be informative. If distribution is too skewed (seems so), I would recommend to dichotomize the variabe into presence vs absence of green spaces in the given buffer. It is very hard for me to believe that green space in such big buffers would be more important for children than green space close to their home/school.**

Indeed, we did not add the 500m because of the skewed distribution. We like the suggestion to dichotomize the greenness exposure based on the presence vs the absence of green space within a 500m buffer. In the figures we do include now the 500m buffer.

We added this in the statistical analysis on page 8, line 232: “*We expressed the estimates for an IQR change in percentage greenness for buffers from 1000 to 5000m. Due to the skewedness of the data for the 500 m radius, we expressed the effect-size for a dichotomized variable representing the presence or absence of green space within this buffer.”*

**8.3 Also, CORINE's minimum mapping unit is 25 ha which means that smaller parks are not present in those dataset. Some Authors even say that CORINE is not exactly applicable if urban areas (e.g.,** [**https://bmcpublichealth.biomedcentral.com/articles/10.1186/1471-2458-12-337**](https://bmcpublichealth.biomedcentral.com/articles/10.1186/1471-2458-12-337)**), which I agree with. Have Authors tried using Landsat-derived vegetation indexes (NDVI, SAVI, EVI, etc.) as a sensitivity analysis? I would be curious to see whether results are similar.**

In this study, we calculated green space (semi-natural-, forested -, blue- and urban green areas and not agricultural areas) based on CORINE Land Cover 1990 (European Environment Agency). Green spaces include city parks, community gardens, sports ﬁelds, as well as natural and forested areas in rural environments. Indeed, CORINE data does not capture small patches of green space and includes only areas that are at least 25 ha. For this reason, green space in buffer sizes smaller than 1000m was not investigated.

On request of the reviewer, we calculated greenness based on a high resolution method. We added to the methods (page 6, line 185): “*Moreover, we calculated residential green space based on a high-resolution (1 m²) land cover dataset which is available, Green Map of Flanders 2012 from the Agency for Geographic Information Flanders (AGIV). We estimated high green (vegetation height higher than 3m), within several radii (2000, 1000, 500, 300, 100, 50m) around the residence.”*

Comparing low-resolution green space calculated by CORINE land cover with specific measures from this high-resolution (1 m²) land cover dataset in an urban area shows that green space (>10m buffer) is significant positive correlated high green (vegetation height higher than 3m) while it is negative correlated with low green (vegetation height lower than 3m) and agricultural area. This gives important insight in what the green of CORINE represents and we now have extended the discussion on this on page 14, line 442: “*However, our results were comparable when we use high resolution maps (S7 Fig)*.”

| **Table Correlation between green space based on CORINE land cover compared to specific measures of green estimated based on the high-resolution dataset** | | |
| --- | --- | --- |
|  | **1000m** | **2000m** |
| **Total Green** | 0.28  <0.0001 | 0.42  <0.0001 |
| **High Green** | 0.48  <0.0001 | 0.50  <0.0001 |
| **Low Green** | -0.056  0.39 | 0.10  0.11 |
| **Agricultural area** | -0.31  <0.0001 | -0.38  <0.0001 |

We added to the results (page 10, line 105): *“Further, we investigated the association between intelligence and residential high green (vegetation height higher than 3m) based on a high-resolution model. Our results (S7 Fig) show that high green is positively associated with verbal and total IQ in large buffer sizes (> 500m).”*

**S7 Fig Estimated change in intelligence quotient (IQ) for an IQR increase in green space (high-resolution model 2012) in a 50-2000m radius around the residence.** Green space is high green (vegetation height higher than 3m, including blue). In twins living in A) an urban (n=236), B) a suburban (n=128), and C) a rural area (n=256). Adjusted for sex, age, parental education, neighbourhood household income, year of IQ test, and zygosity and chorionicity.

**8.4 Finally, since Authors dealt with CORINE-derived green spaces, I would rename the article and use „green spaces" instead of „greenness" throughout the manuscript.**

We changed as requested “greenness” into “green space” throughout the manuscript.

**9. L144-159: What are minimum and maximum scores of the both WISC-R and CBCL tests, in total and of the subscales? This information would be of interest for a reader.**

We added to the methods (page 7, line 206): “*The minimum and maximum scores that can be obtained for total, verbal and performance IQ are 50 and 150 points.* “

We added to the methods (lines 211-216 on page 7): *“Parents were asked to answer each item in 3 levels: 0=not true, 1=sometimes/somewhat true, 2=very true/often true, with higher score indicating more adverse behaviour. The sum of all questions served as a global score for children's problematic behaviour (the total problems behaviour score). Separate scores were calculated for internalizing problems and externalizing problems. CBCL T-scores equal to 64 or higher on the internalizing, externalizing, or total problem scales are considered in the clinical range (Achenbach et al., 2001).”*

Achenbach TM, Rescorla LA. Manual of the ASEBA school-aged forms and profiles. Burlington, VT: University of Vermont, Research Center for Children, Youth, and Families; 2001

**10. L171, L173: Please briefly mention why quadratic year was added to the models.
Have the Authors checked non-linearity of the studied associations somehow? This might be a reason for not seeing some associations. At least, I would recommend to categorize green space variables in tertiles in a sensitivity analysis.**

To capture potential non-linearity of period and time trends on the study outcomes we tested quadratic terms of study year. However, in the models with the changes suggested by reviewers such as removing distance to major road from the main model, the quadratic term was not more significant and removed from the analysis.

In the statistical methods we mention now: “*To capture potential non-linear effects of year of assessment, the quadratic terms of this variable was tested, but did not reach significance. Further, to allow for non‐linearity, green space variables were categorised into tertiles. We constructed scatterplots (Figure 1) and in addition we plotted IQ (total, verbal and performance) for each tertile of green space (3000m buffer). We did not find evidence for deviation from linearity.”* This was added to the methods on page 8, lines 235-239.

We give the figure below for the reviewer information.

**Figure Mean (95% CI) intelligence quotient (IQ) plotted against tertiles of green space in a 3000m around the address at time of the measurement for children residing in an urban area.** A) Total IQ, B) verbal IQ, C) performance IQ. Adjusted for sex, age, parental education, neighbourhood household income, year of IQ test, and zygosity and chorionicity.

**11. L172: Why models were adjusted for distance to major road (and how this major road was defined?)? As a proxy for air pollution and/or noise? In this case, I would suggest to at least look at the effect estimates without this variables (if the Authors believe air pollution and noise are confounders) or even to do mediation analysis (if the Authors think this is mediation).**

We used residential distance to major road as a proxy for traffic related noise and air pollution. Major roads were defined by two types of roads, namely freeways and national roads. We removed the variable distance to major road from our main models and adjusted for distance to major roads and regional background levels of NO_2_ (see answer on question 22) in a sensitivity analysis. This did not result in a considerable change in the interpretation of our findings (S5 Fig).

**S4 Main model additionally adjusted for distance to major road and NO_2_ exposure.** Estimated change in intelligence quotient (IQ) for an IQR increase in green space in a 1000-5000m radius around the residence in children living in an urban (n=236) area. The main model is adjusted for sex, age, parental education, neighbourhood household income, year of IQ test, and zygosity and chorionicity. We additionally adjust for distance to major road and regional background levels of NO_2_ exposure the year before IQ test.

**12. Table 1: How exactly parental education was defined - as the highest education of both parents based on the available information? Please clarify in the text.**

We clarified in the text (page 6, line 166): “*Further we collected information on parental education. The highest educational level of both parents as a proxy of socioeconomic status (SES) was categorized into two groups according to the Belgian education system: (1) no education, primary school, lower secondary education, or higher secondary education, and (2) tertiary education.”*

**13. L203 and Figure 2: The strongest association was actually with 2000m buffer.
To all figures: Please consider using different symbols rather than colours, as many people have problems with differentiating colours. Figure 4 caused me eye pain, for instance.**

*L203 In an urban area, residential greenness within a 3000-4000m buffer is significantly associated with total -, verbal - and performance IQ.*

Indeed, the strongest association was found with a 2000m buffer. We corrected the sentence to (page 9, line 281): *“In urban areas, residential green space within a 1000 to 5000m buffer is significantly associated with total - and performance IQ, while the association between urban greenness and verbal IQ was significant in a 1000 to 4000m buffer and not in* a 500m and 5000m buffer.”

*Figure 4 was adapted and transferred to supplement (S3 Fig):*

**S3 Fig Estimated change in intelligence quotient (IQ) for an IQR increase in green space in a 1000-5000m radius around the residence during pregnancy and in childhood.** Dark blue: Residential green space in childhood at time of IQ measurement (n=236) adjusted for sex, age, parental education, neighbourhood household income, year of IQ test (linear and quadratic), and zygosity and chorionicity. Red: Residential green space during pregnancy (n=216) adjusted for sex, gestational age, birth weight, birth year (linear and quadratic), zygosity and chorionicity, cord insertion, parental education, neighbourhood household income, and smoking during pregnancy.

**14. L228-233: Actually, there is no associations overall, only in urban children. The three sentences should be re-formulated accordingly.**

We clarified (page 11, line 321):” *The key finding of our analysis is that a higher percentage of residential green space is associated with higher intelligence and lower behavioural problems in 7-15 year old children living in urban areas. More particularly, the level of urbanicity modified the association of green space exposure with intelligence and behaviour in childhood*.”

**15. L237: This paper can also be cited:**

[**https://ehp.niehs.nih.gov/doi/full/10.1289/EHP1876?url_ver=Z39.88-2003&rfr_id=ori:rid:crossref.org&rfr_dat=cr_pub%20%200pubmed**](https://ehp.niehs.nih.gov/doi/full/10.1289/EHP1876?url_ver=Z39.88-2003&rfr_id=ori:rid:crossref.org&rfr_dat=cr_pub%20%200pubmed)

*L237 A study in schoolchildren in Barcelona [10] shows that school and total surrounding greenness were associated with enhanced progress in working memory and superior working memory and a greater reduction in inattentiveness.*

We agree that this study is highly relevant and we changed it as follows (page 11, line 330):*” The same investigator found in a subcohort of 253 children, using high-resolution 3D MRIs, several brain regions that had larger volumes in association with higher lifelong exposure to residential surrounding greenness. (Dadvand 2018).”*

**16. L237-243: This is pretty much a repetition of L71-77.**

We added Dadvand et al., 2018 and Browning *et al.,* 2018 to the discussion (page 11, line 328):

“*A study in schoolchildren in Barcelona [10] shows that school and total surrounding greenness were associated with enhanced progress in working memory and superior working memory and a greater reduction in inattentiveness. The same investigator found in a subcohort of 253 children, using high-resolution 3D MRIs, several brain regions that had larger volumes in association with higher lifelong exposure to residential surrounding greenness (Dadvand 2018). In a prospective birth cohort study, Dadvand and colleagues established that higher lifelong residential surrounding greenness was associated with better scores on tests of attention at 4-5 year and 7 year of age [23]. ~~In addition, a positive significant association was noted between surrounding green space of schools in Massachusetts and school-level performance in both English and Math [11].~~ In addition, based on school-level student performance data from 905 public schools in Massachusetts, the second most tree-covered state, collected between 2006 and 2012 (n= 6333), the effect of surrounding greenness on the school performance was investigated. The results showed that children (age 8-9 years) with higher exposure to greenness show better performance in both English and Math [11]. However, this study was replicated in Chicago, in an* area with low tree coverage, *and found no convincing evidence for a positive relationship between residential greenness and academic performance (Browning et al., 2018). The authors suggest that a main reason may be that greenness, the general degree of vegetation, based on NDVI fails to distinguish between vegetation types, such as grasses and trees. In connection to this Kweon et al. found a positive association between tree cover in the school surrounding and academic performance, but grass and shrubs negatively correlate with performance (Kweon et al., 2017). “*

We removed the study of Wu et al. from the introduction and replaced this with (page 4, line 92):

“*In a previous study of 2,593 children attending primary school in Barcelona [10], exposure to surrounding greenness at the home and school area was associated with greater progress in working memory and attention [10]. ~~In addition, based on school-level student performance data from 905 public schools in Massachusettes collected between 2006 and 2012 (n= 6333), the effect of surrounding greenness on the school performance was investigated. The results showed that children (age 8-9 years) with higher exposure to greenness show better performance in both English and Math[11].~~ The literature on green space around schools and academic performance shows mixed results (Browning et al., 2019). Although positive associations were observed for three types of green space: green land cover (45% of the findings), greenness (32%) and tree cover (30%) whereas no positive findings were found for agriculture, grass, or shrub cover.”*

**17. L261: This study should also be cited:** [**https://www.ajpmonline.org/action/showPdf?pii=S0749-3797%2817%2930377-X**](https://www.ajpmonline.org/action/showPdf?pii=S0749-3797%2817%2930377-X)

Feng, X.; Astell-Burt, T. Residential Green Space Quantity and Quality and Child Well-Being: A Longitudinal Study. Am. J. Prev. Med. **2017**, 53, 616–624.

We included more studies on greenspace and behaviour (including the study of Feng *et al*., 2017). Therefore, we completely rewrote this section and replaced it by (page 12, line 364):

“*Our findings show that residential green space is associated with a reduction in total and mostly externalizing problem behaviour (including attention problems and aggressive behaviour) in 7-15 year old children living in a urban area. This in line with other studies on green space using Child Behaviour Checklist (CBCL) to assess behaviour. School children 6-18 years of age living in the highest tertile of greenness had a lower total CBCL score, especially aggressive behaviour and attention problems, compared with children living in the lowest tertile of greenness (Lee et al., 2019). As in our study, the researchers found a slightly stronger association between greenness and externalizing behaviour than with internalizing behaviour (Lee et al., 2019). In urban-dwelling adolescents, exposure to neighbourhood greenspace is associated with reducing aggressive behaviour as reported by parents on the aggression subscale of the CBCL (Younan 2016). A systematic review present evidence for an association between green space exposure and the total difficulties score of the Strengths and Difficulties Questionnaire (SDQ), a measure of general mental health in children (Vanaken et al. 2018). Regarding the different subdomains of the SDQ, five out of six studies documented significant associations with hyperactivity and inattention problems in association with green space exposure (Zach et al., 2016; Amoly et al., 2014; Balseviciene et al., 2014; Markevych et al., 2014; Richardson et al., 2017). A study in 4 to 13-year-old children shows that green space quality and quantity were associated with internalizing and externalizing subscale scores for all age groups (Feng et al., 2017).* “

**18. L266-267: Markevych et al. (ref. 17 in your list) also had urban and suburban-rural composition of the study area, speaking of urbanicity. There are many more studies on other outcomes that investigated effect modification by urbanicity, just from what I can easily remember:** [**https://www.sciencedirect.com/science/article/pii/S1353829218311158**](https://www.sciencedirect.com/science/article/pii/S1353829218311158) **and** [**https://www.mdpi.com/1660-4601/13/3/311/htm**](https://www.mdpi.com/1660-4601/13/3/311/htm).

*266 The majority of studies investigating greenness and health are restricted to urban green and studies comparing urban and rural environments are very limited. A previous study [26] shows that the association between green space and health varied according to the combination of urbanicity and area income deprivation.*

We included more studies investigated effect modification by urbanicity, including the study of Markevynch *et al.* (2014). In addition, we also discussed the disparities observed in urban compared to rural and suburban areas.

We added to the discussion:

On page 11, line 345:

*“Few studies on green space and academic performance have specifically tested for effect-modification by urbanicity. A cross-sectional study in Maryland public schools tested the relationship between five greenspace measures and performance (n=668) around schools and in children's neighborhoods (Browning et al., 2020). Including interaction terms in the models showed no clear effect modification by urbanicity.”*

On page 12, lines 397:

*“The participants in the study of Markevych (2014) were spatially spread over the inner city of Munich and its surrounding areas. The analyses stratified by whether or not a child lived in the inner city, showed that the associations between urban green spaces and lower behavioural problems was slightly stronger for children living in the inner city (n = 782) compared with those living in the surrounding suburban areas (n = 1150), however the interaction-term was not significant.”*

*On page 13, lines 385:*

*“Several reasons can be postulated why no association is found between green space and IQ as well as behavioural problem scores in suburban and rural residents compared to urban residents. First, Markevych et al. (2014) suggest that there is a general lack of green spaces in urban areas, and thus parks play a more important role for urban residents than for those living in suburban or rural areas, in which natural green spaces are more prevalent. A previous study [26] shows that the association between green space and health varied according to the combination of urbanicity and area income deprivation. A possible explanation is that high-income residents in suburban and rural areas have their own domestic gardens, and municipal green space is thus space is thus less important to them [26]. Finally, rural areas might have fewer everyday destinations (e.g. grocery store, post office and pharmacy) within walking distance, resulting in more car dependency and less active transportation (Markevych et al., 2017). Therefore, urban residents may have more benefits of green space in their neighbourhood.”*

**19. L270-272: In this context, I would especially encourage the Authors to consider some satellite-derived vegetation index, which would capture all vegetation, not only green spaces min 25 ha in area.**

*L270-272: A possible explanation is that high-income residents in suburban and rural areas have their own domestic gardens, and municipal green space is thus less important to them [26].*

See answer question 8.3. above.

**20. L273-300: This section is too long considering that the Authors did not attempt to investigate any of the mechanisms by mediation analyses. I would suggest condensing this part.**

We condensed this part of the discussion (page 1, line 396): “*The underlying pathways responsible for the positive effect of residential greenness exposure on intelligence is not fully understood, but several mechanisms are considered. First, green spaces provide environmental benefits (e.g. reducing exposure to air pollution, noise and heat). Moreover, green spaces encourage health-promoting activities and facilitating social cohesion. A third potential domain linking greenspace to health is the ability of greenspace to restore capacities (e.g. attention restoration and physiological stress recovery). (Markevych et al., 2017)”*

**21. L305-307: This is not true. Landsat 4-5 TM satellite images are available since early 1980s, and they could have been used in this study in addition to CORINE 1990. Among the limitations, which are clearly more than two: poor quality land cover data to derive green spaces data, as specified before, unavailability of full residential history and time-activity pattern, absence of mediation analysis. Even effect modification by SES was not checked.**

*L305-307: Second, the twins were born from 1981 till 1991, however to determine residential greenness during pregnancy we used data from 1990 as no earlier satellite data were available.*

The CORINE land cover data were from 1990. No earlier satellite data on land-use were available. However, green spaces calculated based on CORINE land cover is highly correlated even over a decade. See table below.

| **Table Correlation coefficient between residential green space based on CORINE land cover 1990 and green space based on CORINE land cover 2000 (n=620)** | | | | | |
| --- | --- | --- | --- | --- | --- |
|  | **1000** | **2000** | **3000** | **4000** | **5000** |
| Residential green space based on CORINE 2000 | 0.99  <0.0001 | 0.99  <0.0001 | 0.99  <0.0001 | 0.99  <0.0001 | 1.00  <0.0001 |

As requested we extended in the revised discussion on the limitation paragraph which reads now as follows (page 14, lines 423): *“*Third, no information was available on, time activity patterns such as time spend outdoors, and on possible mediators between green space and intelligence.*”*

Finally, we added, sensitivity analysis on high-resolution map see answer previous question 8.3.

The reviewer mentioned that information on full residential history is not available but this information was available. We clarified this now, see in this regard also our answer on previous question 7 (L133-134).

Further as suggested we now checked potential effect modification by SES. Based on parental education we stratified the children residing in an urban area in to groups low parental education (n=82) and high parental education (n=154).

We added to the results (page 10, 301): *“We performed stratified analysis to study the potential effect-modification by parental education. Patterns were similar according the parental educational level but overall we found a tendency of more pronounced associations between residential greenness and IQ outcomes in urban children of parents with higher educational background (S6 Fig).”*

**S6 Fig Effect modification socioeconomic status.** Estimated change in intelligence quotient (IQ) for an IQR increase in green space in a 1000-5000m radius around the residence in children living in an urban. Based on parental education we stratified the children residing in an urban area in to groups low parental education (n=82) and high parental education (n=154). Adjusted for sex, age, parental education, neighbourhood household income, year of IQ test, and zygosity and chorionicity.

**22. L309: Additional adjustment for distance to major road does not exclude confounding by air pollution, as distance to major road is just a mere proxy. I wonder if real air pollution data were not available. For example, there are ozone and NO2 maps from the ELAPSE project for the year 2000, and the estimates can be back-extrapolated. In any case, I would at least soften the language in this part.**

*L309: An important strength of our study is that we excluded potential confounding by socioeconomic indicators and traffic related air pollution.*

*“We calculated the regional background levels of NO_2_ for the residential address during childhood using a kriging interpolation method (Jacobs et al. 2010; Janssen et al. 2008) that uses land cover data obtained from satellite images. This model provides interpolated NO_2_ values from the Belgian telemetric air quality networks in 4 × 4 km grids. We calculated individual NO_2_ concentrations (micrograms per cubic meter) one year before the measurement.”* This was added to the methods page 7 line 191.

We adjusted the main models for NO_2_ exposure and distance to major road in a sensitivity analysis. This did not result in a considerable change in the interpretation in the aforementioned estimates (S5 Fig). This was added to the results page 10 line 300.

**S5 Fig Main model additionally adjusted for distance to major road and NO_2_ exposure.** Estimated change in intelligence quotient (IQ) for an IQR increase in green space in a 1000-5000m radius around the residence in children living in an urban (n=236) area. The main model is adjusted for sex, age, parental education, neighbourhood household income, year of IQ test, and zygosity and chorionicity. We additionally adjust for distance to major road and regional background levels of NO_2_ exposure the year before IQ test.

**23. More effort should be put into explaining why green space was not important for suburban and rural residents compared to urban residents. Also, why no associations were observed for internalizing problems? Why not for all buffers associations were present? In my view, Discussion needs to be shorten in some parts and elaborated in others.**

Several reasons can be postulated why no association is found between green space and IQ in suburban and rural residents compared to urban residents. This was added to the discussion. See answer on previous question 18 (L266-267).

Our findings show that residential green space is associated with a reduction in total and externalizing problem behaviour are in line with other studies finding an association between green space and behaviour problems such as attention problem and aggressive behaviour in children living in an urban area (see answer on question 17).

We also explain differences observed between the radius distances around the residential address. We added to the discussion (page 14, line 418)*:” The association between green space and the outcomes IQ and behaviour was investigated in several radius distances around the residential address. We found that the positive impact of green in an urban setting for both IQ and behavioural scores depended on the percentage green within 2000 and 3000m. It was unfeasible to choose buffers smaller than 500m given the resolution of CORINE land cover data.”*

We condensed the part explaining underlying mechanism of green space. See answer on question 20 (273-300).

Reviewer #3:

Thank you for inviting me to review the manuscript “The importance of residential greenness on intellectual and behavioral development in children living in urban versus rural environments” for PLOS Medicine. The main objective is to analyze the association between environmental green space and intelligence and behavior in a study that includes urban and rural environments across east Flanders in Belgium. I think findings do contribute to existing knowledge nowadays on this topic. However, I have some commentaries about this paper.

**This paper has two major contributions; firstly, previous studies investigate the association between greenness and cognition and behavior and this one analyze intelligence assessed by Wechsler Intelligence Scale for Children (WISC-R). The existing literature shows its beneficial effects on specific cognitive and behavioral functions, instead of total IQ. That is why this is one of the strengths of the work. However, the affirmation in line 92 of “the effect on intelligence is unknown “sounds if they were completely separate and independent constructs.**

*L92 The previous research mentioned above investigated greenness in association with cognitive and behavioural aspects, however the effect on intelligence is unknown.*

*We replaced this sentence in the introduction (page 4, line 113): “The previous research mentioned above investigated green space in association with cognitive and behavioural aspects, in connection to this we investigate the association between green space and intelligence.”*

**On the other hand, being the second strength of the study the fact that they analyze differences in two different areas, urban and rural, and taking into account that there are not many evidences in analyzing the associations between greenness and rural areas, more information in this sense it should be added in the introduction.**

We added to the introduction*(page 4, line 110):* “*Few studies on green space and cognition (Browning et al., 2020). or behaviour (Lee 2019, Markevych 2014) have investigated effect modification by urbanicity but did not find statistically significant differences by urbanization of residence.”*

*In the discussion we elaborate on* the disparities observed in urban compared to rural and suburban areas:

On page 11, line 345:

*“Few studies on green space and academic performance have specifically tested for effect-modification by urbanicity. A cross-sectional study in Maryland public schools tested the relationship between five greenspace measures and performance (n=668) around schools and in children's neighbourhoods (Browning et al., 2020). Including interaction terms in the models showed no clear effect modification by urbanicity.”*

On page 12, lines 397:

*“The participants in the study of Markevych (2014) were spatially spread over the inner city of Munich and its surrounding areas. The analyses stratified by whether or not a child lived in the inner city, showed that the associations between urban green spaces and lower behavioural problems was slightly stronger for children living in the inner city (n = 782) compared with those living in the surrounding suburban areas (n = 1150), however the interaction-term was not significant.”*

*On page 13, lines 385:*

*“Several reasons can be postulated why no association is found between green space and IQ as well as behavioural problem scores in suburban and rural residents compared to urban residents. First, Markevych et al. (2014) suggest that there is a general lack of green spaces in urban areas, and thus parks play a more important role for urban residents than for those living in suburban or rural areas, in which natural green spaces are more prevalent. A previous study [26] shows that the association between green space and health varied according to the combination of urbanicity and area income deprivation. A possible explanation is that high-income residents in suburban and rural areas have their own domestic gardens, and municipal green space is thus space is thus less important to them [26]. Finally, rural areas might have fewer everyday destinations (e.g. grocery store, post office and pharmacy) within walking distance, resulting in more car dependency and less active transportation (Markevych 2017). Therefore, urban residents may have more benefits of green space in their neighbourhood.”*

I have some other minor remarks:

**1) Page 3. In the first paragraph, evidences of the impact of air pollution and neurodevelopment are introduced. However, the relationship between the main construct, greenness, and air pollution is not presented. Reduction of air pollution is one of the beneficial consequences of the exposition of green spaces but is not the only one, and there are more biopsychosocial mechanisms that try to explain the beneficial effects. Exposition to green spaces has environmental benefits because it reduces air pollution, air pollution, or even heat. But this idea is explained later on, and it results confusing.**

We explained possible underlying pathways later in the discussion. We agree that we better replace this to the introduction.

Therefore, we expanded the section in the introduction (page 3, line 74):

“*Green space provide environmental benefits by reducing air and noise pollution. Higher residential green space is associated with lower ambient air pollution [27] and noise exposure [28]. Both have been associated with diminished cognitive development [29-32]. Furthermore, from a health perspective, green space promotes physical activity and stress reduction [9] [33, 34]. Apart from the promotion of physical activity, green space in the living environment could also lead to more social contacts [36]. In addition, higher levels of neighbourhood green space were associated with significantly lower levels of symptoms of depression, anxiety and stress. [40] Besides self-reported stress, also salivary cortisol, an indicator of chronic stress, is negatively associated with exposure to greenness in the living environment [41]. Higher residential green space during pregnancy is even associated with longer telomere length in placental tissue, an important marker of ageing [42]. Finally, according to the biodiversity hypothesis of health, the reduced contact with a natural environment and biodiversity is associated with adverse implications for the human microbiome and its immunomodulating capacity [43]. Microbial input from the environment may drive brain regulation and is a possible major component of the beneficial effect of greenness [44]. Several animal studies suggest that the human microbiome may have profound effects on brain development and linked the gut microbiota to neurodevelopmental processes and behavior [45-47]. Natural space-induced environmental biodiversity has been related to enriched skin and gut microbiota that in turn has been shown to improve brain development, probably through regulation of immune response [50].”*

**2) Page 3 lines 61-62: “environmental factors may impact these performances and neuroinflammation” neuroinflammation is not explained and developed.**

We adjusted the sentence to (page 3, line 64): *“Environmental factors* ***such as air pollution are associated with neuroinflammation*** *and may impact these performances in healthy children (Calderón-Garcidueñas 2008). Children residing in a highly polluted urban environment exhibited deficits in fluid intelligence, memory, executive functions and also impairments in subscales of intelligence quotient (IQ), relative to children living in a less polluted urban environment (Calderón-Garcidueñas 2008).”*

**3) Page 5: In relation to WISC-R: I would add more information about such as number of items, max and min…On the other hand, who did it administer? Was it administered by a unique trained person or more than one? in the second case information about inter-evaluator reliability. In line 150 “the total scores of the subscales and..” it is not clear that the subscale are verbal and performance.**

The WISC-R contains 12 subscales. We mention in the methods (page 7, line 200): *“The verbal subscales are Information (INF), Similarities (SIM), Arithmetic (ARI), Vocabulary (VOC), Comprehension (COM) and Digit Span (DS). The performance subscales are Picture Completion (PC), Picture Arrangement (PA), Block Design (BD), Object Assembly (OA), Coding (COD) and Mazes (MAZ).”*

We added to the methods (page 7, line 206): “*The minimum and maximum scores that can be obtained for total, verbal and performance IQ are 50 and 150 points.”*

Twin IQ was evaluated by trained observers. We did not find any observer effect on IQ outcomes. For the reviewer information we additionally adjusted for evaluator of the IQ test. The figure is shown below.

We clarified the sentence in the methods (page 7, line 205):” The scores on the subscales are standardized for age and added up to Verbal (VIQ), Performance (PIQ) and Total Intelligence Quotients (TIQ). In this study, the verbal, performance and total IQ scores were analysed.”

**Figure** **Main model additionally adjusted for evaluator IQ test.** Estimated change in intelligence quotient (IQ) for an IQR increase in green space in a 1000-5000m radius around the residence in children living in an urban (n=236) area. The main model is adjusted for sex, age, parental education, neighbourhood household income, year of IQ test, and zygosity and chorionicity. We additionally adjust for the evaluator of the IQ test.

**4) Page 6 line 152: Achenbach Child behavior Checklist (CBCL) is not in the reference list. Is there a Dutch validated version?**

We added the reference to the reference list:

“Achenbach T. Integrative guide for the 1991 CBCL/4–18, YSR, and TRF profiles. Burlington: Department of Psychiatry, University of Vermont; 1991.”

Yes, there is a Dutch validated version. We added this information to the methods (page 7, line 208) *“Behaviour was assessed by the Achenbach Child Behaviour Checklist (CBCL). This checklist was developed by Achenbach (1991) to examine the extent to which children have behavioural and emotional problems as perceived by their parents (Achenbach et al., 1991). Good reliability and validity have been demonstrated for the Dutch CBCL (Verhulst et al., 1996).”*

Verhulst FC, van der Ende J, Koot HM (1996), *Dutch Manual for the CBCL/4-18.* Rotterdam: Afdeling Kinder- en Jeugdpsychiatrie, Sophia KinderziekenhuidAcademisch Ziekenhuis Rotterdam, Erasmus Universiteit Rotterdam

**5) Figure 1. A) B) and C) are not in the title of each one of the figure (total intelligence quotient, verbal and performance intelligence quotient. In this figure the reason why two correlations are made is not explained (urban area n=230 and 234; suburban na= 126 and 128 and rural only with n=252).**

We added **“**A”, “B” and “C” as a tittles of figure 1 A, B, and C respectively.

For robustness we excluded 8 potential data points with green space >20%. Correlation coefficients are shown for data points in figure (excluding points with greenness >20%, n=8) and for all data points, showing similar findings.

We adapted the legend of the figure:

| **Urban area** | n = 232; r = 0.21; *p* = 0.001 | n = 232; r = 0.22; *p* = 0.001 | n = 232; r = 0.14; *p* = 0.03 |
| --- | --- | --- | --- |
|  | n = 236; r = 0.24; *p* = 0.0002 | n = 236; r = 0.22; *p* = 0.0006 | n = 236; r = 0.19; *p* = 0.003 |
| **Suburban** | n = 126; r = 0.08; *p* = 0.35 | n = 126; r = 0.12; *p* = 0.18 | n = 126; r = 0.03; *p* = 0.73 |
|  | n = 128; r = 0.11; *p* = 0.22 | n = 128; r = 0.14; *p* = 0.12 | n = 128; r = 0.06; *p* = 0.53 |
| **Rural area** | n = 254; r = -0.09; *p* = 0.17 | n = 254; r = -0.05; *p* = 0.39 | n = 254; r = -0.10; *p* = 0.12 |
|  | n = 256; r = -0.11; *p* = 0.08 | n = 256; r = -0.06; *p* = 0.31 | n = 256; r = -0.13; *p* = 0.04 |

**Figure 1 The association between intelligence and residential green space is modified by the degree of urbanicity (p for interaction = <0.0001).** Intelligence in association with green space in a 3000m radius around the current residence in twins living in an urban (n=232), suburban (n=126), and a rural area (n=254). Data points (n = 8) with green space >20% were excluded from the figure for visibility of the figure. A) Total intelligence quotient, B) verbal intelligence quotient, C) performance intelligence quotient. Correlation coefficients are shown for data points in figure (excluding points with green space >20%) and for all data points.

**6) Page 7 line 215: Prenatal greenness exposure data collection is not described in Methods and it is not mentioned neither in Introduction. That is why the analysis between greenness during pregnancy with intelligence is not expected. This information should be added in the respective sections in order to understand this result and the paragraph of the Discussion, page 9 line 254.**

*L214 Besides greenness at the residential address in childhood, we also observed a significant association between greenness during pregnancy with intelligence in children living in an urban area (Figure 4).*

*L254 Besides greenness at the residential address in childhood, we also observed a significant association between greenness during pregnancy with intelligence in children living in an urban area. Till now, only one study in 1,312 women and their children investigated surrounding green spaces at the residential address at birth. It shows that exposure to higher levels of surrounding green spaces is associated with better early childhood cognition at the age of two years [25].*

We moved this figure to the supplement (S3 Fig).

We calculated green space at the residential address during pregnancy and during childhood. The method is similar. We describe in the methods (page 6, line 181)*: “*Semi-natural-, forested -, blue- and urban green areas (green space) in several radius distances (5000, 4000, 3000, 2000, and 1000m) around the residential address during childhood and during pregnancy were estimated based on CORINE Land Cover 1990 (European Environment Agency). Green space does not include agricultural areas.”

We added to the introduction (page 4, line 121): “*We hypothesize that the level of urbanicity can modify the outcome on intelligence and behaviour in children associated with green space exposure in early-life.”*

**7) Page 8 line 228: The first sentence refers to a strength/contribution of the work. I suggest to put it on later on in the Discussion, after explaining the main conclusions. Furthermore, the same idea is repeated in the second paragraph “by our knowledge this is the first study investigating the association between residential greenness and intelligence in children”.**

*L228 Till now no studies on intelligence and residential greenness were reported. The key finding of our analysis is that residential greenness is associated with a higher intelligence and a reduction in behavioural problems in 7-15 year old children.*

We removed this first sentence of the discussion and we kept the sentence in the second paragraph *(line 325 on page 11):* “*By our knowledge this is the first study investigating the association between residential greenness and intelligence in children.”*

We added later in the discussion (line 442 on page 14): “*An important contribution of this paper is that to our knowledge this is the first study on intelligence and residential greenness.”*

**8) In Discussion the results related to behavioral components are not commented. An association is observed in total and externalizing problems (including attention and aggressive behavior). There are evidences in this sense that should be discussed.**

We discussed our findings in the discussion (page 12, line 364):

“*Our findings show that residential green space is associated with a reduction in total and mostly externalizing problem behaviour (including attention problems and aggressive behaviour) in 7-15 year old children living in a urban area. This in line with other studies on green space using Child Behaviour Checklist (CBCL) to assess behaviour. School children 6-18 years of age living in the highest tertile of greenness had a lower total CBCL score, especially aggressive behaviour and attention problems, compared with children living in the lowest tertile of greenness (Lee et al., 2019). As in our study, the researchers found a slightly stronger association between greenness and externalizing behaviour than with internalizing behaviour (Lee et al., 2019). In urban-dwelling adolescents, exposure to neighbourhood greenspace is associated with reducing aggressive behaviour as reported by parents on the aggression subscale of the CBCL (Younan 2016). A systematic review present evidence for an association between green space exposure and the total difficulties score of the Strengths and Difficulties Questionnaire (SDQ), a measure of general mental health in children (Vanaken et al. 2018). Regarding the different subdomains of the SDQ, five out of six studies documented significant associations with hyperactivity and inattention problems in association with green space exposure (Zach et al., 2016; Amoly et al., 2014; Balseviciene et al., 2014; Markevych et al., 2014; Richardson et al., 2017). A study in 4 to 13-year-old children shows that green space quality and quantity were associated with internalizing and externalizing subscale scores for all age groups (Feng et al., 2017).* “

**9) Before publication a revision of English should be done by a native speaker.**

We carefully checked the language and we would be happy to do further language editing by a native speaker upon acceptance of our manuscript.
